# Visual short-term memory impairments in presymptomatic familial Alzheimer's disease: A longitudinal observational study

**DOI:** 10.1016/j.neuropsychologia.2021.108028

**Published:** 2021-11-12

**Authors:** Ivanna M. Pavisic, Jennifer M. Nicholas, Yoni Pertzov, Antoinette O'Connor, Yuying Liang, Jessica D. Collins, Kirsty Lu, Philip S.J. Weston, Natalie S. Ryan, Masud Husain, Nick C. Fox, Sebastian J. Crutch

**Affiliations:** a Dementia Research Centre, Department of Neurodegenerative Diseases, UCL Institute of Neurology, London, UK; b UK Dementia Research Institute at University College London, London, UK; c Department of Medical Statistics, London School of Hygiene and Tropical Medicine, London, UK; d Department of Psychology, The Hebrew University of Jerusalem, Israel; e Nuffield Department of Clinical Neuroscience, University of Oxford, UK; f Department of Experimental Psychology, University of Oxford, UK

**Keywords:** Familial Alzheimer's disease, Alzheimer's disease, Preclinical Alzheimer's disease, Visual short-term memory, Estimated symptom onset, VSTM, visual short-term memory, AD, Alzheimer's disease, FAD, familial Alzheimer's disease, PMC, presymptomatic mutation carrier, EYO, estimated years to/from symptom onset, AYO, actual years to/from symptom onset, NART, National Adult Reading Test

## Abstract

Visual short-term memory (VSTM) deficits including VSTM binding have been associated with Alzheimer's disease (AD) from preclinical to dementia stages, cross-sectionally. Yet, longitudinal investigations are lacking. The objective of this study was to evaluate VSTM function longitudinally and in relation to expected symptom onset in a cohort of familial Alzheimer's disease. Ninety-nine individuals (23 presymptomatic; 9 symptomatic and 67 controls) were included in an extension cross-sectional study and a sub-sample of 48 (23 presymptomatic carriers, 6 symptomatic and 19 controls), attending two to five visits with a median interval of 1.3 years, included in the longitudinal study. Participants completed the *“*What was where?” relational binding task (which measures memory for object identification, localisation and object-location binding under different conditions of memory load and delay), neuropsychology assessments and genetic testing. Compared to controls, presymptomatic carriers within 8.5 years of estimated symptom onset showed a faster rate of decline in localisation performance in long-delay conditions (4s) and in traditional neuropsychology measures of verbal episodic memory. This study represents the first longitudinal VSTM investigation and shows that changes in memory *resolution* may be sensitive to tracking cognitive decline in preclinical AD at least as early as changes in the more traditional verbal episodic memory tasks.

## Highlights

• VSTM function was investigated in presymptomatic and symptomatic FAD carriers.
• PMCs showed faster decline in VSTM function (target localisation) than controls.
• Target localisation accuracy decreased with proximity to expected symptom onset.
• “What was where?” may be sensitive to tracking preclinical cognitive decline.

## Credit author statement

Ivanna M. Pavisic: Conceptualization, Methodology, Investigation; Writing-Original draft; Jennifer M. Nicholas: Methodology, Formal Analysis, Writing-Review & Editing; Yoni Pertzov: Conceptualization, Methodology, Software, Writing-Review & Editing; Antoinette O'Connor: Project administration, Investigation, Writing-Review & Editing, Funding acquisition; Yuying Liang: Methodology, Data Curation, Investigation, Writing-Review & Editing; Jessica D. Collins: Investigation, Writing-Review & Editing; Kirsty Lu: Data Curation, Writing-Review & Editing; Philip S. J. Weston: Project administration, Investigation, Writing-Review & Editing, Funding acquisition; Natalie S. Ryan: Investigation, Writing-Review & Editing; Masud Husain: Conceptualization, Methodology, Writing-Review & Editing; Nick C. Fox: Conceptualization, Methodology, Writing-Review & Editing, Funding acquisition; Sebastian J. Crutch: Conceptualization, Methodology, Writing-Review & Editing, Supervision, Funding acquisition.

## 1 Introduction

Progressive episodic memory impairment is a central, defining feature of Alzheimer's disease (AD) (Dubois et al., 2007; McKhann et al., 1984). Deficits in short-term memory (STM), the ability to temporarily maintain information over seconds (Atkinson and Shiffrin, 1968, 1971), have been relatively less well studied. Classically, STM has been tested using ‘span’ measures where participants are asked to remember a string of stimuli (Groeger et al., 1999). Although such quantal (discrete) measures have been fundamental to developing our understanding of memory function, they are not as sensitive to detect changes in memory *resolution* due to the binary nature of responses measured (correct *vs* incorrect recall). In 2014, Ma and colleagues (Ma et al., 2014) proposed a new approach to study the resolution with which items are retained, arguing that just because an individual fails to recall an item correctly this does not imply they had no memory of it at all. Delayed-reproduction tasks (e.g. (Peich et al., 2013; Pertzov et al., 2012, 2013)) rely on remembering a feature and reproducing the exact stored features after a retention period using a *continuous analogue* response space (Bays et al., 2009; Gorgoraptis et al., 2011; Liang et al., 2016). In recent studies, delayed-reproduction tasks have been reported to be more sensitive than conventional span measures of STM, especially in clinical populations (Zokaei et al., 2015).

The concept of ‘preclinical AD’ continues to evolve and is subject to debate, but current clinical criteria at least on a research basis, allow for it to be diagnosed in asymptomatic individuals without evidence for objective cognitive decline (Sperling et al., 2011) but with accumulation of β-amyloid (Aβ) (Jack and Holtzman, 2013). Developing a better understanding of the preclinical changes of AD and improving methods for early detection may offer the best chance for therapeutic success, before irreversible neuronal loss has occurred.

One important line of research has suggested that the ability to bind object features together in visual short-term memory (VSTM) is critically affected in AD (Della Sala et al., 2012; Parra et al. 2009, 2010a, 2011, 2011; Pavisic et al., 2020a). Interest in these tasks increased when studies suggested impairments could be detected at preclinical stages of the condition, more sensitively than other traditional memory measures (Parra et al., 2010b). A study by Liang and colleagues found deficits for object-location binding and localisation of the target position in presymptomatic familial Alzheimer's disease (FAD) carriers, in the most challenging task conditions (highest load and longest delay for object-location binding and highest-load across delays for localisation of the target position (Liang et al., 2016)). FAD is an autosomal dominant condition caused by mutations in either presenilin 1 (*PSEN1*), presenilin 2 (*PSEN2*) or amyloid precursor protein (*APP*) (Ryan et al., 2016) and its pathogenic mutations in these genes are nearly 100% penetrant (Ryman et al., 2014). FAD shares many features (i.e. clinical, radiological and histopathological) with sporadic AD (Rossor et al., 1996; Ryan and Rossor, 2010) and the age at onset in FAD is reasonably similar between family members, making this cohort particularly valuable to the study of preclinical stages of AD (Ryman et al., 2014).

In light of these findings, a number of questions remain unanswered:

1. Are the cross-sectional preclinical deficits in VSTM also reflected in longitudinal task performance?;
2. Given that an individual's expected age at symptom onset may be estimated from their parental onset, what is the relationship between an individual's VSTM performance and proximity to expected years to onset (EYO) at the time of testing?; And finally, for comparison:
3. Is longitudinal cognitive decline in presymptomatic and symptomatic mutation carriers seen in other more traditional neuropsychology tasks?

We wished therefore first to extend the work of Liang and colleagues (Liang et al., 2016) in a larger sample and secondly to explore how VSTM in both presymptomatic and symptomatic FAD mutation carriers, changed with EYO. Finally, for comparison we evaluate longitudinal decline in traditional neuropsychology tasks. To our knowledge, no other study has examined VSTM functions longitudinally in a preclinical cohort such as FAD.

## 2 Methods

### 2.1 Study design and participants

Participants were recruited from the ongoing longitudinal FAD study at the Dementia Research Centre, University College London, which receives referrals from across the UK, if they had an autosomal dominant family history of AD and a known pathological mutation in *PSEN1* or *APP* genes in at least one affected family member. Healthy individuals (without a family history of AD) were also recruited to the study from our research database. Inclusion criteria also required participants to have normal or corrected-to-normal visual acuity and colour vision and ≥70% average accuracy in identification performance at baseline visit (see (Liang et al., 2016)).

Mutation analysis was carried out using Sanger sequencing (Janssen et al., 2003; Ryan et al., 2016). Genetic results were available for all at-risk individuals, on either a clinical or a research basis. Research genetic results were only shared with the statistician involved in the study and were not disclosed to the participants or to other researchers who remained blind to whether presymptomatic individuals were mutation carriers or non-carriers.

Consequently, the study included symptomatic carriers, presymptomatic carriers and controls: symptomatic individuals were mutation carriers who had cognitive symptoms consistent with AD; presymptomatic individuals were mutation carriers who had not developed symptoms and who scored zero on the Clinical Dementia Rating (CDR) scale (Morris, 1993) and control participants consisted of both non-carriers (at-risk individuals who tested negative for pathological mutations) and healthy individuals (from our research database). As per Liang and colleagues, we used EYO as an approximation of how far individuals (presymptomatic and symptomatic) were from symptom onset (Liang et al., 2016). This was based on an individuals' age at the time of assessment subtracted from the age at which their affected parent developed symptoms (Bateman et al., 2012; Ryman et al., 2014) with a positive value indicating years from/post onset and a negative value indicating years to/pre onset. Similar to previous FAD studies (Weston et al., 2020), we carried out an exploratory analysis whereby PMCs were separated into those furthest away and those closest from expected onset using the median value of EYO of PMCs in our dataset (8.5 years before onset). This resulted in the following four groups based on a participant's status at the baseline assessment: symptomatic carriers; ‘early’ presymptomatic mutation carriers (PMCs) (more than 8.5 years from expected onset), ‘late’ PMCs (less than 8.5 years from expected onset) and controls. This was done recognizing that subtle cognitive changes occur even at presymptomatic stages of FAD (e.g. (O'Connor et al., 2020)) and that by isolating PMCs closest to onset, subtle cognitive deficits would be more pronounced than in PMCs furthest away from expected onset.

In addition, we also considered how performance varied continuously with a) EYO (for all FAD carriers: symptomatic and presymptomatic) and b) actual years to/from symptom onset (AYO) for symptomatic carriers (N = 6) and PMCs who converted into symptomatic carriers throughout the study (N = 3). Actual age at onset was defined as the age at which progressive symptoms of FAD were first noticed by the individual or someone who knew the patient well.

The cross-sectional analysis included 99 individuals: 67 controls (16 non-carrier siblings) and 32 mutation carriers, 9 of whom were symptomatic. Differences between our cross-sectional study and Liang and colleagues (Liang et al., 2016) were: the addition of n = 17 at-risk (mutation carriers and non-carriers) individuals; n = 1 symptomatic carrier and the exclusion of n = 1 at-risk individual (see Supplementary Materials, Fig. e1, for details). Note that mutation status of these at-risk individuals is not disclosed to prevent unblinding of genetic status.

The longitudinal analysis included 48 participants who attended between 2 and 5 visits (median 3), at intervals ranging from 0.5 to 3.9 years (median 1.3): 19 controls (12 non-carrier siblings) and 29 mutation carriers, 6 of whom were symptomatic from the first assessment. (Mean follow-up time: controls = 2.8 [SD 1.7] years, range = 1–6; early PMCs = 3.7 [1.7] years, range = 1–6 years; late PMCs = 3.4 [1.7] years, range = 1–6; symptomatic carriers = 2.6 [0.7] years, range = 2–4)).

All subjects provided written informed consent to participate. The study was approved by The National Hospital for Neurology and Neurosurgery and Institute of Neurology Joint Research Ethics Committee (subsequently, National Research Ethics Service Committee, London Queen Square, Research Ethics Committee ref 11/LO/0753).

### 2.2 Protocol

The study protocol included a clinical and neuropsychological assessment and the *“*What was where?” VSTM experiment (Pertzov et al., 2012). Detailed interviews were conducted with individuals at-risk of FAD and their close informants to assess for the presence of cognitive or behavioural symptoms attributable to AD. AD was diagnosed in accordance with the Dubois criteria (Dubois et al, 2007, 2010). Folstein's mini-mental state examination (MMSE) (Folstein et al., 1975), the CDR (Morris, 1993) and Hospital Anxiety and Depression scale (Zigmond and Snaith, 1983) were administered.

The neuropsychological test battery included measures of several cognitive domains: episodic memory (recognition memory test (RMT) for words and faces (Warrington, 1996)); working memory (digit span (Wechsler, 1987)); intellectual function (Wechsler Abbreviated Scale of Intelligence (WASI) (Wechsler, 1999)); executive function (Stroop (1935)); confrontational naming (graded naming test (McKenna and Warrington, 1983); vocabulary (British picture vocabulary scale (BPVS) (Dunn and Dunn, 2009)); arithmetic (Graded Difficulty Arithmetic Test (GNT) (Jackson and Warrington, 1986)), visual perception (object decision test from the visual object and space perception (VOSP) battery (Warrington and James, 1991)); processing speed (digit symbol test (Wechsler and De Lemos, 1981)) and estimated premorbid intelligence (the National Adult Reading Test) (NART) (Law and O'Carroll, 1998; Nelson, 1991) (Table 1).

**Table 1:** Baseline demographics, neuropsychology and VSTM performance by participant group for N = 99.

	Controls (N = 67)	Early PMCs (N = 12)	Late PMCs (N = 11)	Symptomatic carriers (N = 9)
Demographics				
Sex: N (%) Male	34 (50.7)	3 (25.0)	7 (63.6)	6 (66.7)
Age (yrs)	39.4 (8.1)	34.8 (6.4)	37.0 (5.0)	48.1 (9.8)*
EYO (yrs)	NA	−12.9 (4.7)	−5.8 (1.8)	3.0 (4.1)
AYO (yrs)	NA	NA	NA	3.1 (4.0)
Education (yrs)	15.4 (2.7)	14.3 (2.5)	13.3 (2.5)*	13.9 (2.9)
MMSE	29.5 (0.8)	29.3 (0.9)	29.5 (0.8)	25.1 (3.7)**
CDR global	0.0 (0.0)	0.0 (0.0)	0.0 (0.0)	0.6 (0.2)**
Anxiety	6.1 (3.8)	7.9 (4.6)	3.9 (3.9)*	7.0 (4.5)
Depression	3.2 (2.8)	2.9 (4.0)	1.3 (1.6)*	2.4 (2.1)
Neuropsychology tests				
Performance IQ	110.5 (16.3)	106.0 (15.7)	101.4 (10.1)	100.4 (12.1)
Verbal IQ	109.9 (14.9)	96.1 (15.1)*	95.4 (13.5)**	99.4 (18.8)
Arithmetic total/24	16.7 (6.8)	13.9 (5.0)	14.3 (4.6)	10.3 (5.8)**
RMT faces	41.1 (7.2)	41.0 (4.2)	43.8 (4.5)	40.3 (3.7)
RMT words	47.0 (5.0)	48.7 (2.2)	46.5 (2.8)	35.3 (10.0)**
Digit span forwards/8	7.1 (1.2)	6.8 (1.0)	7.4 (1.1)	6.0 (1.5)*
Digit span backwards/7	5.2 (1.2)	5.7 (1.3)	5.4 (1.1)	4.3 (1.6)
BPVS	142.5 (8.8)	135.0 (14.4)**	139.8 (10.1)	135.9 (11.8)
GNT/30	20.9 (4.6)	17.8 (5.8)	19.2 (5.4)	18.8 (7.2)
NART/50	31.8 (8.9)	24.1 (8.6)**	27.7 (10.7)	25.4 (13.2)
VOSP OD/20	18.0 (2.8)	17.8 (1.8)	18.3 (1.3)	17.6 (1.5)
Stroop (s)	50.3 (14.0)	45.8 (12.2)	52.6 (14.1)	78.2 (22.4)**
VSTM performance				
Identification (% correct)				
Overall	91.6 (4.8)	90.2 (6.3)	92.0 (3.9)	81.9 (5.0)**
Localisation error (deg)				
Overall	4.4 (1.3)	4.5 (1.3)	4.6 (1.1)	7.8 (1.8)**
Swap error (%)				
Overall	10.6 (5.3)	11.7 (4.7)	10.2 (5.9)	22.6 (8.1)**
Block 1, 1s delay	12.0 (8.4)	12.4 (9.2)	9.9 (5.0)	21.2 (12.6)
Block 1, 4s delay	13.2 (8.7)	18.7 (9.2)	15.0 (10.8)	23.2 (18.0)*

Unadjusted mean values are given with SD unless otherwise stated. SD = standard deviation; NA = not applicable; PMC = presymptomatic mutation carrier; EYO = estimated years to/from symptom onset (a negative value indicates a younger age than their estimated age at symptom onset); AYO = actual years to/from onset (positive values indicate years post onset); Anxiety and depression scores from HADS = hospital anxiety and depression scale; IQ = intelligence quotient; MMSE = mini mental state examination; CDR = clinical dementia rating scale; RMT = recognition memory test; GNT = graded naming test; VOSP OD = object decision from the visual object and space perception battery. Digit spans forwards and backwards are taken from the WMS-R **=** Wechsler Memory Scale. Neuropsychology data were available at baseline for: 64 participants for performance IQ, verbal IQ; 98 for arithmetic total, GNT, NART, VOSP; 99 for RMT faces, RMT words, digit span forwards, digit span backwards; 71 for BPVS; and 78 for Stroop (s). Bold = significant; *: the difference between the patient group and controls for that variable was significant at *p* < 0.05; **: the difference between the patient group and controls for that variable was significant at *p* < 0.01.

**Fig. 1:**
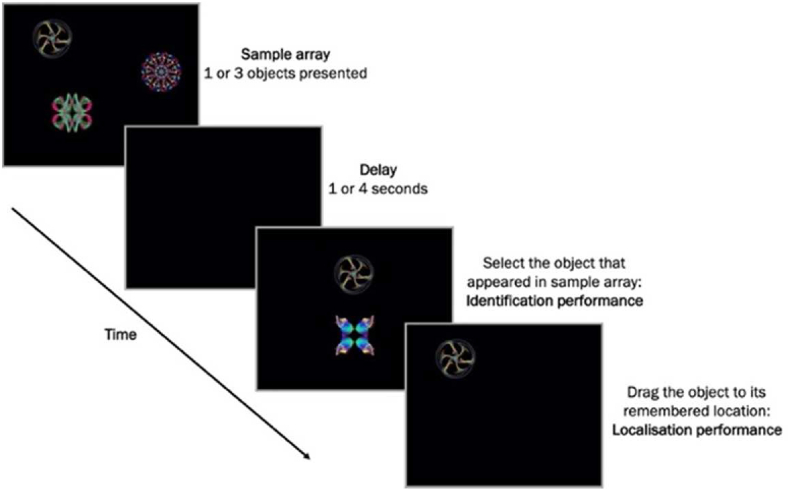
**Schematic of “What was there?”** (adapted from (Liang et al., 2016) under the terms of the Creative Commons Attribution License (CC BY)).

“What was where?” has been described in previous publications (Liang et al., 2016; Pertzov et al., 2012). A depiction of the task is shown in Fig. 1. Participants sat approximately 42 cm in front of an interactive touch-sensitive screen (Dell Inspiron One 2320) with a 1920 × 1080-pixel matrix corresponding to approximately 62 × 35° of visual angle. In each trial, participants viewed 1 or 3 fractal objects, each randomly located on the screen and were asked to remember both the object's identity and their locations. A blank screen was then displayed for a 1 or 4seconds (s) duration, followed by a test array in which two fractals appeared along the vertical meridian. One of these was in the previous memory array (the target fractal) whereas the other one was a foil (distractor). The foil was not an unfamiliar object, but was part of the general pool of fractal images presented throughout the experiment. All objects including the foils were drawn from a pool of 60 fractals that were used across the experiment (rendered using http://sprott.physics.wisc.edu/fractals.htm). Participants were required to select the fractal they remembered from the memory array and drag it to its location. This provided a continuous measure of localisation error. Each participant performed a practice block of 10 trials (not included in the analysis) followed by two test blocks each consisting of 10 trials with 1 fractal and 40 trials with 3 fractals, with a balanced number of trials with 1s or 4s delay between memory and test arrays.

In this paper, findings focus on three outcomes which were included in the previous cross-sectional study (Liang et al., 2016):

• **Identification performance:** proportion of trials where the correct object was chosen.
• **Localisation error:** the distance (in degrees of visual angle) between the centre of the target object once placed in its remembered location and its true (original) location in the memory array (only correctly identified objects).
• **Swap errors:** the percentage of correctly identified objects placed within 4.5 deg eccentricity of other fractals in the original array-3-item condition only (object-location binding). In accordance with previous studies (Liang et al., 2016; Pertzov et al., 2012), a threshold of 4.5 deg was used as objects were never presented less than 9 deg from each other in the memory array and therefore an object could not be swapped with more than one object.

Liang and colleagues also examined the “Nearest item control (NIC)”: an index of localisation precision regardless of object identity, calculated as the distance between the centre of the target object once placed in its remembered location and the centre of the nearest location in the memory array –whichever item that was, i.e., it is agnostic to the identity of the nearest fractal. It provided a measure of localisation error discounting the effects of swap errors for the 3-items condition only (Liang et al., 2016; Pertzov et al., 2013). Results for this outcome are provided in the Supplementary Materials (section 2.2).

### 2.3 Statistical analysis

Due to a skewed distribution the absolute localisation error was log transformed and proportion of swap errors was square root transformed before analysis.

#### 2.3.1 Cross-sectional analysis

As an extension study, data was initially analysed cross-sectionally. Baseline demographics and neuropsychology scores were compared between controls and each of symptomatic carriers, early PMCs and late PMCs using ANOVA, or Kruskal-Wallis test where the distribution of the variable was skewed. Fishers’ exact test was used to compare the sex distribution between the groups.

VSTM performance at the baseline visit was compared between controls and each of symptomatic carriers, early PMCs and late PMCs using logistic regression models for object identity and linear regression model for all other measures. Robust standard errors were used to account for repeated measures. Models were adjusted for delay (1 *vs* 4s), block (1 *vs* 2), number of items (1 *vs* 3, where relevant), sex, age at baseline, and NART at baseline. Interaction tests were used to examine whether group differences in performance, varied by delay, block and number of items.

#### 2.3.2 Longitudinal analysis

In order to evaluate VSTM function longitudinally, change over time in VSTM was investigated in three ways: **i)** comparison of rate of change in VSTM performance (for each metric) between controls and each of symptomatic carriers, early PMCs, late PMCs (with groups defined by their status at baseline). Longitudinal change in object identity was analysed using a mixed effects logistic regression model and analysis of the other VSTM outcomes used a linear mixed effects model. Models were adjusted for delay, block, number of items (where relevant), sex, age at baseline, and NART at baseline and interaction tests were used to examine whether group differences in rates of change in performance varied by delay, block and number of items. **ii)** examination of the association between VSTM performance and EYO as a continuous measure (in presymptomatic and symptomatic mutation carriers) after adjusting for healthy ageing. This was done by including mutation carrier status, EYO, EYO squared (in mutation carriers only) and age at visit as predictors in the model. Including the control data allowed estimation of the effect of age at visit across both controls and mutation carriers. The rationale for including age at visit was to ensure that any difference observed in VSTM performance with increasing EYO could be attributed to EYO rather than ageing, since those closer to onset also tend to be of older age. This analysis using EYO as a continuous measure was included to address limitations associated with categorising the EYO measurement (Altman and Royston, 2006). **Iii)** examination of association between VSTM performance and AYO as a continuous measure in the FAD participants where this was known (symptomatic carriers at baseline (N = 6) and late PMCs who became symptomatic during the study-‘converters’: N = 3) after accounting for healthy ageing as for the EYO analysis. The rationale for this analysis was that actual, rather than expected, years to onset would provide a more precise estimation of how VSTM function varies with proximity to onset. As for approach ‘‘i)’, object identity was analysed using a mixed effects logistic regression model and analysis of the other VSTM outcomes used a linear mixed effects model for approaches ‘ii)’ and ‘iii)’. In approaches ‘ii)’ and ‘iii)’, for each model the predicted mean difference was calculated for controls and by EYO or AYO in the carriers, setting age and NART at the average of the sample and for an equal balance of sexes and task conditions and differences between the mean for mutation carriers and the mean in controls were also calculated by EYO and AYO. Furthermore, models were adjusted for delay, block, number of items (where relevant), sex and NART at baseline and interaction tests were used to examine whether the association with EYO or AYO (respectively) differed by delay, block and number of items.

Finally, in order to evaluate whether cognitive decline in presymptomatic and symptomatic mutation carriers was seen in other more traditional neuropsychology tasks, longitudinal change in neuropsychology performance was compared between controls and each of symptomatic carriers, early PMCs and late PMCs. Mixed effects linear regression was used for analysis of WASI verbal IQ, WASI performance IQ, arithmetic, BPVS, GNT, NART, and Stroop. A mixed effects logistic regression model was used for RMT words, RMT faces and VOSP. Mixed effects ordinal logistic regression model was used for digit span forwards and digit span backwards. All models were adjusted for sex, age at baseline, and NART at baseline.

For all analysis statistical significance was set at *p* < 0.05 and analysis performed on Stata v.14 or later.

See the Supplementary Materials for further details on the statistical methods (section 1.2).

## 3 Results

### 3.1 Cross-sectional analysis for N = 99

#### 3.1.1 Demographics and traditional neuropsychology

Baseline characteristics of the groups are presented in Table 1. Sixty-seven controls and 32 carriers completed the “What was where?” task cross-sectionally. Early PMCs were on average 12.9 years away from their expected onset and compared to controls were on average younger (t (95) = -1.89, *p* = 0.062), and had lower scores in: verbal IQ (t (60) = -2.55, *p* = 0.013), BPVS (U = 113, *p* = 0.004) and NART measures (U = 204.5, *p* = 0.006). Late PMCs were on average 5.8 years before expected onset, and compared to controls had lower education (U = 183.5, *p* = 0.021), lower baseline anxiety (U = 219*, p* = 0.034) and depression scores (U = 205, *p* = 0.018) and had significantly lower scores for verbal IQ (t (60) = -2.78, *p* = 0.007) but similar scores on remaining measures. Symptomatic carriers were on average 3.0 years after expected onset and as expected were older than controls (t (95) = 3.11, *p* = 0.026), had lower MMSE (U = 65, *p* < 0.001) and significantly worse scores on neuropsychology tasks including arithmetic (t (94) = -2.74, *p* = 0.007), RMT for words (U = 79, *p* = 0.001), digit span (U = 158*, p* = 0.015) and Stroop (U = 39, *p* < 0.001) (Table 1). Additionally, the global CDR score, was indicative of cognitive impairments consistent with AD (at a relatively early stage: mean = 0.6 (SD 0.2), range = 0.5–1, Table 1).

Baseline characteristics of the participants included in the longitudinal study (N = 48) are given in the Supplementary Materials (Table e1).

#### 3.1.2 VSTM performance

VSTM performance was significantly worse with higher-memory load (3 *vs* 1 item) (odds ratio (OR) for correct identification = 0.20 [95% CI 0.15, 0.28], Z = −9.86, *p* < 0.001; localisation error = 2.82 times higher [2.65, 3.00], t (97) = 33.1, *p* < 0.001). Longer delay (4 *vs* 1s) was also associated with worse identification performance (OR = 0.82 [CI 0.71, 0.95], Z = −265, *p* = 0.008) and localisation performance (localisation error = 26.9% greater [21.9%, 32.1%], t (97) = 11.73, *p* < 0.001) but did not affect swap proportion (difference in √swap error proportion = 0.002 [-0.026, 0.030], t (97) = 4.74, *p* = 0.255).

**Fig. 2:**
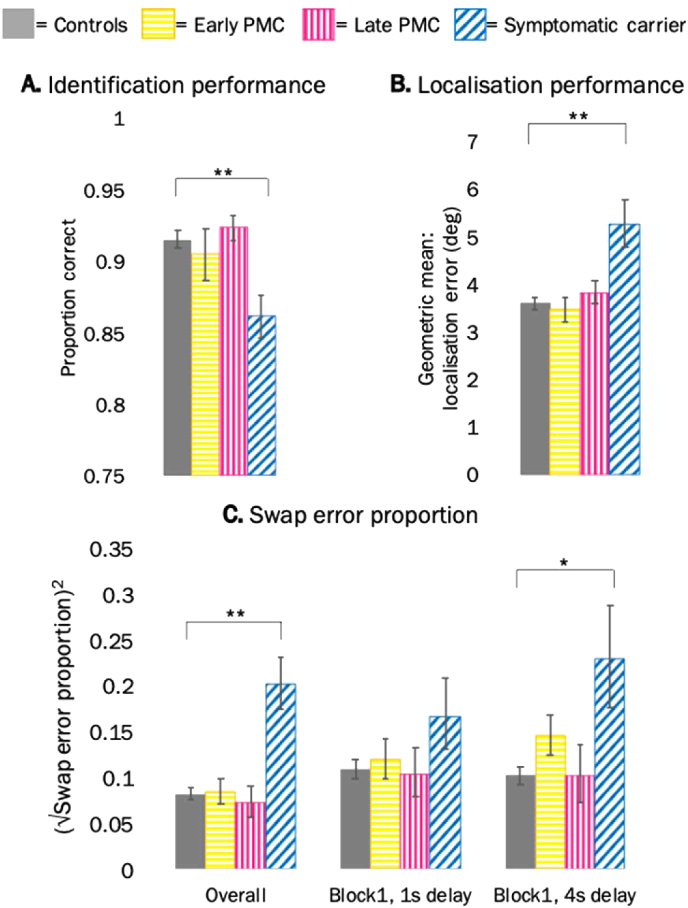
**Cross-sectional adjusted mean performance by group (from model adjusted for age, sex and NART). A.** Identification performance (across all conditions); **B.** Localisation error (across all conditions); **C.** Swap error proportion across all conditions and by delay in block 1. Error bars show ± standard error of the mean. PMC = presymptomatic mutation carrier. * = significant at *p* < 0.05; ** = significant at *p* < 0.01.

Symptomatic carriers had 44.0 [95% CI 25.4, 56.7] % lower odds of correctly identifying the target (Z = −4.08, *p* < 0.001), 46.0 [20.1, 77.5] % greater localisation error (t (97) = 3.85, *p* < 0.001) and made a greater proportion of swap errors (difference in √swap error proportion = 0.162 [0.095, 0.231], t (97) = 4.74, *p* < 0.001) in comparison to controls (also see Table 1 for unadjusted mean values). There were no significant differences between early PMCs and controls or late PMCs and controls (Table 1, Fig. 2). There was no evidence for an interaction between group and delay or group and number of items in identification (interaction tests across groups: delay Χ^2^ (3) = 4.34, *p* = 0.227; items (Χ^2^ (3) = 1.96, *p* = 0.580) or localisation (interaction tests across groups: delay F (3,97) = 1.08, *p* = 0.362; items F (3,97) = 1.17, *p* = 0.327) performance. However, there was an interaction between delay and the proportion of swap errors (interaction test across groups: F (3,97) = 2.90, *p* = 0.039), whereby symptomatic carriers showed larger differences compared to controls in the long-delay (difference in √swap error proportion = 0.203 [0.121, 0.285]; t (97) = 4.89; *p* < 0.001) than the short-delay (difference in √swap error proportion = 0.123 [0.058, 0.187]; t (97) = 3.78; *p* < 0.001) (interaction tests for delay: symptomatic carriers: F (1,97) = 8.27, *p* = 0.005; early PMCs: F (1,97) = 1.71, *p* = 0.194; late PMCs: F (1,97) = 0.01, *p* = 0.904).

Although there was no significant interaction of group with block (interaction tests across groups: identification Χ^2^ (3) = 3.09, *p* = 0.378; localisation F (3,97) = 1.92, *p* = 0.131; swaps F (3,97) = 2.06, *p* = 0.110), we investigated performance in the first block by delay, following Liang and colleagues finding of a significantly higher proportion of swap errors in the PMC group than controls, in the first block long-delay condition (Liang et al., 2016). As was seen in the analysis combing both blocks, in block 1 symptomatic carriers showed larger differences compared to controls in the long-delay condition (difference in √swap error proportion = 0.159 [0.039, 0.279]; t (97) = 2.62; *p* = 0.010) than the short-delay (difference in √swap error proportion = 0.079 [-0.02, 0.179]; t (97) = 1.55; *p* = 0.125) (interaction test for delay in block 1, symptomatic carriers: F (1,97) = 8.20, *p* = 0.005). No significant differences emerged at a presymptomatic level for either delay in block 1 (Table 1, Fig. 2).

Taken together, the cross-sectional findings presented here do not replicate entirely Liang and colleagues’ study (Liang et al., 2016). Specifically, while symptomatic participants showed a poorer performance for all metrics in both studies, PMCs (early or late) did not show evidence for a greater swap error proportion in the longest-delay condition as reported previously (Liang et al., 2016). See the Supplementary Materials (section 2.4, Fig-e.5) for a direct comparison between the new participants and the cohort previously published by Liang and colleagues (Liang et al., 2016) and the Discussion for possible reasons for these differences.

### 3.2 Longitudinal analysis for N = 48

Forty-eight individuals completing at least two annual visits were included in the longitudinal analysis: 19 controls; 20 individuals who remained PMCs throughout the duration of the study: 12 early PMCs, 8 late PMCs; 3 converters-participants who were late PMCs at baseline but had symptoms at their last follow-up visit and 6 symptomatic carriers.

Considering all visits together, longer delay (1 *vs* 4s); higher memory load (3 *vs* 1 item); and block 1 (*vs* block 2) had significant effects on VSTM metrics resulting in worse: localisation, identification and swap error performance (greater error, poorer performance, see the Supplementary Materials, section 2.3, for the effect size of delay, memory load and block on each VSTM metric).

#### 3.2.1 Rates of change in VSTM function

##### 3.2.1.1 Identification performance

Throughout the course of the study, there was little change over time in identification performance within controls and PMCs (Z = −0.11, *p* = 0.913; early PMCs: Z = 0.19, *p* = 0.850; late PMC: Z = −1.23, *p* = 0.217), whereas performance for symptomatic carriers decreased over time (Z = −2.53, *p* = 0.011) (see Table 2 for the percentage odds of correct identification over time within each group).

**Table 2:** Rates of change in VSTM function per year. The first row indicates the change over time within each group (change/year). The second row compares the rate of change for each patient group to that of controls (difference in change/year).

Change per year	Controls (N = 19)	Adjusted mean [95% CI] Group difference [95% CI] (control as reference)		
		Early PMCs (N = 12)	Late PMCs (N = 11)	Symptomatic carriers (N = 6)
Identification performance: % change in odds of correct response				
Overall	−0.4 [-8.0, 7.8]	0.8 [-6.8, 8.9]	−5.6 [-13.8, 3.4]	−15.3 [-25.5, -3.7]*
	NA	1.2 [-9.2, 12.8]	−5.2 [-16.1, 7.2]	−14.9 [-26.8, -1.1]*
Localisation error: change in % error				
Overall	0.4 [-2.1, 3.1]	0.3 [-2.4, 3.1]	4.1 [0.9, 7.4]*	7.0 [0.6, 13.8]*
	NA	−0.1 [-3.8, 3.7]	3.6 [-0.4, 7.9]	6.5 [-0.4, 13.9]
3-items	0.4 [-2.2, 3.1]	1.4 [-1.5, 4.3]	4.0 [0.7, 7.5]*	7.3 [0.5, 14.6]*
	NA	0.9 [-3.0, 5.0]	3.6 [-0.7, 8.1]	6.9 [-0.5, 14.7]
3-items, 1s	0.8 [-2.2, 3.9]	0.0 [-3.3, 3.4]	1.4 [-2.3, 5.3]	9.9 [1.7, 18.8]*
	NA	−0.8 [-5.2, 3.8]	0.6 [-4.1, 5.6]	9.0 [0.3, 18.5]*
3-items, 4s	0.0 [-3.0, 3.1]	2.7 [-0.7, 6.2]	6.9 [2.9, 11.0]**	4.8 [-3.4, 13.6]
	NA	2.7 [-1.9, 7.4]	6.9 [1.8, 12.2]**	4.7 [-3.9, 14.2]
1-item	0.6 [-3.2, 4.4]	−3.3 [-7.3, 0.8]	4.4 [-0.5, 9.5]	5.5 [-4.7, 16.9]
	NA	−3.9 [-9.1, 1.7]	3.8 [-2.3, 10.3]	5.0 [-5.9, 17.0]
1-item, 1s	0.9 [-3.1, 5.1]	−4.6 [-8.8, -0.2]*	1.7 [-3.4, 7.0]	8.1 [-3.3, 20.8]
	NA	−5.5 [-11.0, 0.4]	0.7 [-5.6, 7.5]	7.1 [-4.9, 20.5]
1-item, 4s	0.2 [-3.8, 4.3]	−2.0 [-6.3, 2.4]	7.2 [1.8, 12.8]**	3.0 [-7.7, 15.0]
	NA	−2.2 [-7.9, 3.9]	7.0 [0.2, 14.2]*	2.8 [-8.6, 15.7]
Swap error: change in √proportion				
Overall	−0.001 [-0.014, 0.013]	−0.010 [-0.026, 0.006]	0.001 [-0.018, 0.019]	−0.016 [-0.043, 0.011]
	NA	−0.009 [-0.030, 0.012]	0.001 [-0.022, 0.024]	−0.015 [-0.045, 0.014]
Block 1, 4s	−0.014 [-0.035, 0.005]	−0.011 [-0.036, 0.013]	0.014 [-0.014, 0.041]	−0.017 [-0.059, 0.026]
	NA	0.004 [-0.028, 0.036]	0.029 [-0.005, 0.063]	−0.002 [-0.049, 0.045]

Adjusted mean difference in rate of change per year by group and compared to controls. CI= Confidence intervals; NA = not applicable; PMC = presymptomatic mutation carrier. Bold = significant; *: significant at *p* < 0.05. **: significant at *p* < 0.01; % change in odds calculated as (odds ratio-1)*100.

**Fig. 3:**
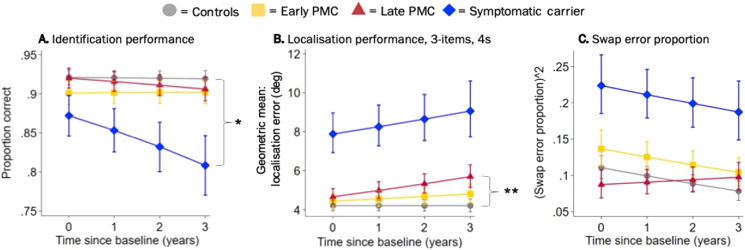
**Longitudinal adjusted estimated mean performance by group (from model adjusted for age at baseline, sex and NART). A.** Identification performance (across all conditions). **B.** Localisation error performance for the 3-item, 4s delay condition. **C.** Swap error performance (across all conditions). PMC = presymptomatic mutation carrier. Error bars indicate ± standard error by time from baseline visit. * = the rate of change between groups was statistically significant at *p* < 0.05 (control as reference); ** = the rate of change between groups was statistically significant at *p* < 0.01 (control as reference).

There was no significant difference in the rate of change of identification performance between either PMC group and controls (early PMCs: Z = 0.22, *p* = 0.830 *vs* the rate of change of controls, late PMCs: Z = −0.85, *p* = 0.395 *vs* the rate of change of controls, see Table 2 for effect sizes), while symptomatic carriers showed a faster decline in identification performance over time (Z = −2.10, *p* = 0.036 *vs* rate of change in controls, with 42.8 [2.5, 66.4] % lower odds of correct identification than controls at baseline decreasing to 64.7 [3.7, 80.2] % lower by year 3, Fig. 3A).

There was no significant interactions on rates of change between group and item number, delay length or block so this metric was not investigated further (interaction test for change over time across all three groups: item: Χ^2^ (3) = 2.64, *p* = 0.451; delay Χ^2^ (3) = 2.08, *p* = 0.557; block: Χ^2^ (3) = 2.90, *p* = 0.408).

##### 3.2.1.2 Localisation performance

Localisation performance generally stayed the same throughout the course of the study for controls (Z = 0.34, *p* = 0.737) and early PMCs (Z = 0.22*, p* = 0.826), whereas localisation error increased over time for late PMCs (Z = 2.55*, p* = 0.011) and symptomatic carriers (Z = 2.13*, p* = 0.033) reflecting a decrease in performance over time (see Table 2 for the adjusted percentage changes in localisation error over time within each group).

Considering all task conditions together, late PMCs and symptomatic carriers showed a trend towards a faster rate of decline in localisation performance compared to controls (late PMCs: Z = 1.74, *p* = 0.082 *vs* the rate of change of controls and symptomatic carriers: Z = 1.84, *p* = 0.066 *vs* the rate of change of controls). No differences in the rate of change were observed between early PMCs and controls (Z = −0.07, *p* = 0.946) (see Table 2 for effect size differences between each patient group and controls).

Both item number and delay length but not block, had a significant effect on differences in performance between groups, with differences from controls larger for longer delay and in the three item condition (overall interaction test for means across all three groups: items: Χ^2^ (6) = 38.46, *p* < 0.001; delay: Χ^2^ (6) = 20.99, *p* = 0.002; block Χ^2^ (6) = 7.71, *p* = 0.260).

There was a significant interaction between delay and group in the rate of change (interaction test for change over time across all three groups: Χ^2^ (3) = 8.57, *p* = 0.036), whereby for the late PMC group in the 4s delay, but not 1s delay condition (interaction test for change over time in late PMCs: Χ^2^ (1) = 6.13, *p* = 0.013), late PMCs showed significantly greater increase in localisation error over time than was seen in the controls (4s, 1-item: Z = 2.02, *p* = 0.043 and 4s, 3-items: Z = 2.67, *p* = 0.008, see Table 2 for effect sizes). This interaction with delay was not seen for the other patient groups (interaction test for change over time: early PMCs: Χ^2^ (1) = 2.40, *p* = 0.123; symptomatic carriers: Χ^2^ (1) = 0.72, *p* = 0.396) and differences in rate of change between groups did not depend on the number of items or block (interaction test for change over time across all three groups: items Χ^2^ (3) = 3.96, *p* = 0.266; block Χ^2^ (3) = 2.50, *p* = 0.474), emphasizing that decline in localisation performance for late PMCs was specific to longer delays. This meant that a difference in localisation error between late PMCs and controls was apparent from 2 years after baseline, with the greatest difference in the 3-items, 4s delay condition (difference 11.0 [-10.0, 36.8] % at baseline, increasing to 35.4 [5.4, 73.8] % at 3 years) (Fig. 3B). The early PMC group did not show differences from controls in the rate of change in localisation error in any condition (Table 2). Symptomatic carriers generally had a faster increase in localisation error than controls, but this only reached statistical significance in the 3-items, 1s delay condition (Z = 2.02, *p* = 0.043, see Table 2 for effect sizes).

##### 3.2.1.3 Swap error performance

Swap error performance for all groups, generally stayed the same throughout the course of the study (controls: Z = −0.08*, p* = 0.937; early PMCs: Z = −1.20, *p* = 0.231; late PMCs: Z = 0.07, *p* = 0.943 and symptomatic carriers: Z = −1.18*, p* = 0.237, see Table 2 for changes in √swap error proportion over time within each group).

There was no difference in rate of change in swap error performance over time between either PMC groups and controls (early: Z = −0.86, *p* = 0.389 *vs* the rate of change of controls, late: Z = 0.10, *p* = 0.917 *vs* the rate of change of controls, see Table 2 for effect size differences). Although symptomatic carriers made a greater proportion of swap errors than controls overall (test for mean difference in performance between groups: Χ^2^ (2) = 20.47, *p* < 0.001; difference in √swap error proportion at baseline = 0.182 [0.093, 0.239], and at visit 3 = 0.135 [0.043, 0.227]), there was no difference in the rate of change compared to controls (Z = −1.02, *p* = 0.309, Fig. 3C) (see Table 2 for effect size differences between each patient group and controls).

While there was only weak evidence that differences between groups in change over time in swaps were influenced by block and delay (interaction test for change over time across all three groups: block: Χ^2^ (3) = 6.59, *p* = 0.086; delay Χ^2^ (3) = 0.62, *p* = 0.089), we specifically examined the 4s delay of block 1, following Liang and colleagues finding of higher swap errors in PMCs in this condition (Liang et al., 2016). There was a trend for a greater increase in swap error proportion over time for late PMCs compared to controls (Z = 1.65, *p* = 0.099 *vs* the rate of change of controls, see Table 2 for effect size), however this effect did not reach statistical significance. No difference in the rate of change *vs* controls was observed for early PMCs (Z = 0.21, *p* = 0.830). Despite having a higher proportion of swaps in the 4s delay of block 1 (test for mean difference in performance between groups: Χ^2^ (2) = 15.32, *p* < 0.001; difference in √swap error proportion at baseline = 0.177 [0.086, 0.264], and at visit 3 = 0.172 [0.054, 0.289]), symptomatic carriers showed no difference in rate of change compared to controls in this condition either (Z = −0.07, *p* = 0.946) (see Table 2 for effect size differences).

#### 3.2.2 Relationship between VSTM performance and proximity to symptom onset

##### 3.2.2.1 Identification performance

**Fig. 4:**
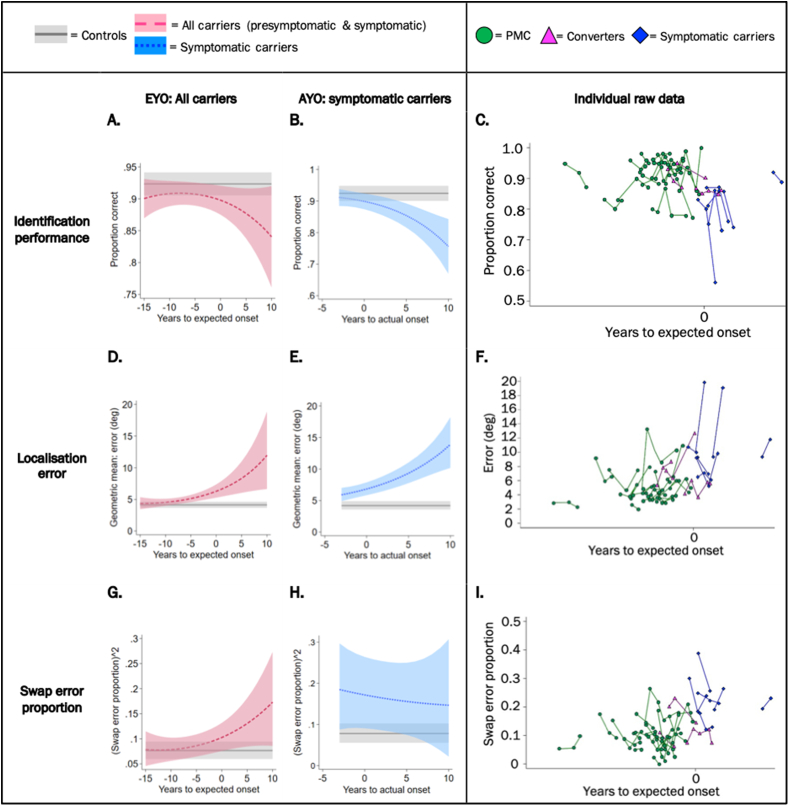
**Relationship between VSTM performance and proximity to symptom onset.** Identification performance is presented across all task conditions; localisation error specifically for the 3-items, 4s delay condition (where the association was strongest) and swap error proportion across delays (by definition only assessed in the 3-items condition). Panels **A., B., D., E., G.** and **H.** show the predicted mean of each VSTM metric (from model adjusted for age, sex and NART) against EYO or AYO. Shaded area indicates 95% confidence intervals. Panels **C**., **F.** and **I.** shows the unadjusted raw data plotted against EYO for each VSTM metric with visits marked as dots and connected for each participant; note there is no scale on the x-axes to preserve participant anonymity. Converters are PMCs who transitioned into a symptomatic stage at their last visit. PMC = presymptomatic mutation carrier. EYO = estimated years to/from symptom onset; AYO = actual years to/from symptom onset.

There was no significant association between identification performance and EYO within FAD carriers (symptomatic and presymptomatic combined, Χ^2^ (2) = 4.24, *p* = 0.120, Fig. 4A–C). Nonetheless, identification performance significantly decreased with AYO in the subgroup analysis of symptomatic carriers and converters (Χ^2^ (2) = 15.78, *p* < 0.001) (Fig. 4B). The association with EYO did not differ by number of items (interaction test: Χ^2^ (2) = 2.42, *p* = 0.298), delay length (interaction test: Χ^2^ (2) = 0.70, *p* = 0.705) or block (interaction test: Χ^2^ (2) = 1.12, *p* = 0.573).

##### 3.2.2.2 Localisation performance

Localisation error significantly increased with EYO in FAD mutation carriers (Χ^2^ (2) = 7.46, *p* = 0.024). There was a significant interaction with item number (interaction test: Χ^2^ (2) = 27.97, *p* < 0.001) and delay length (interaction test: Χ^2^ (2) = 13.60, *p* = 0.001) such that the localisation deficit associated with closer proximity to onset was greater with high load and long delay (i.e. when the memory demands were greatest), but there was no interaction with block (interaction test: Χ^2^ (2) = 2.54, *p* = 0.280). Results were therefore examined by item and delay. There was a significant increase in localisation error with less years to estimated onset for PMCs carriers (or more years post onset for symptomatic carriers) in both 3-item conditions (1s delay: Χ^2^ (2) = 6.64, *p* = 0.036; 4s delay: Χ^2^ (2) = 12.94, *p* = 0.002) but not in the 1-item conditions (1s Χ^2^ (2) = 2.42, *p* = 0.298; 4s Χ^2^ (2) = 4.31, *p* = 0.116). The association was thus strongest in the 3-items, 4s delay condition (Fig. 4D), where a significant difference in mean localisation error between FAD carriers (presymptomatic and symptomatic) and controls was observed from 6 years before expected onset (20.1 [5.5, 41.0] %, Z = 2.62, *p* = 0.024).

Localisation error also significantly increased with AYO within symptomatic carriers and converters (Χ^2^ (2) = 30.15, *p* < 0.001) (Fig. 4E).

##### 3.2.2.3 Swap error proportion

There was no significant association between swap error proportion and EYO within FAD carriers (Χ^2^ (2) = 4.20, *p* = 0.123, Fig. 4G) nor with AYO in the symptomatic group with converters (Χ^2^ (2) = 0.29, *p* = 0.863, Fig. 4H). The association with EYO did not differ by delay length (interaction test: Χ^2^ (2) = 3.75, *p* = 0.153) or block (interaction test: Χ^2^ (2) = 3.58, *p* = 0.167).

#### 3.2.3 Longitudinal change of participants on traditional neuropsychology

Following our findings of a faster rate of decline in localisation performance, we considered rates of change in traditional neuropsychology tasks.

**Fig. 5:**
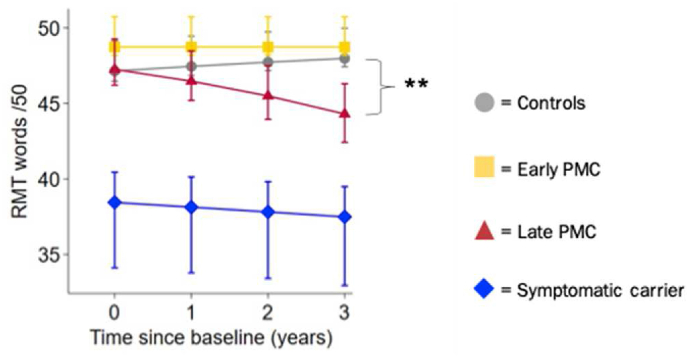
**Longitudinal estimated mean performance for RMT for words by group (from model adjusted for age at baseline, sex and NART).** PMC = presymptomatic mutation carrier; RMT = recognition memory test. Error bars indicate ± standard error by time from baseline visit. * = the rate of change between groups was statistically significant at *p* < 0.05 (control as reference).

A significant difference between late PMCs and controls on the RMT words was observed approximately 1 year later than the presymptomatic changes observed in localisation performance (i.e. from 3 years after baseline), with 35.0 [45.6, 22.2] % greater rate of decline per year (Z = −4.71, *p* < 0.001, Fig. 5).

A significant difference between controls and early PMC group was seen for RMT faces but in the opposite direction to that expected (early PMCs: 13.7 [1.0, 28.0] % greater *increase* in performance per year, Z = 2.12, *p* = 0.034). No further significant group differences emerged at a presymptomatic level. While verbal and performance IQ measures showed lower values for PMCs at baseline, there was no evidence for a faster rate of decline compared to controls (VIQ: early PMCs: 0.9 [-1.1, 2.9] points per year, Z = 0.85, *p* = 0.398; late PMCs: 0.0007 [-2.3, 2.3], Z = −0.00, *p* = 1.000; PIQ: early PMCs: 0.07 [-1.5, 1.6] points per year, Z = 0.09, *p* = 0.930; late PMCs: 0.8 [-2.6, 1.0], Z = −0.91, *p* = 0.363). Symptomatic carriers had a greater rate of decline than controls in: performance IQ (−3.9 [-6.1, −1.7] points per year, Z = −3.45, *p* < 0.001); arithmetic (−1.5 [-2.7, 0.3] points per year, Z = −2.51, *p* = 0.012) and digit span backwards (66% greater decline per year, OR = 0.34 [0.13,0.91], Z = −2.16, *p* = 0.031).

## 4 Discussion

### 4.1 Summary of findings

The aim of this study was to investigate VSTM function over time using the “What was where?” task in a preclinical AD cohort like FAD. More specifically, we investigated a) differences in the rate of change between symptomatic and presymptomatic carriers compared to controls; b) how VSTM varied continuously with proximity to onset and c) whether longitudinal decline was also seen in more traditional measures of neuropsychology. The main findings were that ‘late’ PMCs (within 8.5 years of estimated onset) had a significantly faster rate of decline in localisation performance, which is in agreement with previous reports from our centre of impairment of PMCs on this measure using the same task. However, unlike the previous report (Liang et al., 2016) PMCs did not show a significant difference in the swap error proportion metric – a binary measure of misbinding. Importantly, differences in localisation performance from controls were observed in PMCs at least as early as changes in traditional neuropsychology measures of verbal episodic memory. Localisation performance was also the only VSTM metric to show a significant association with EYO with strongest effects observed in long delay conditions up to 6 years prior to estimated symptom onset. Other important findings include: symptomatic carriers showing a faster rate of decline in identification and localisation performance (though localisation effects were only found significant in one condition – 3-items, 1s – most likely due to small numbers in this group) and identification performance decreasing with increasing AYO.

Taken together, the finding of localisation deficits in FAD carriers – especially in those who were presymptomatic – indicates that the “What was where?” task may be sensitive in tracking preclinical decline.

### 4.2 Preferential effect on localisation performance: what is this metric measuring?

Relational binding in the “What was where?” task is conventionally measured using the ‘swap error proportion’ metric. In this approach, if the fractal is placed within 4.5 deg of another fractal in the memory array, it is considered ‘swapped’. While this pre-defined threshold measures misbinding as a proportion of error (in comparison to change-detection paradigms which evaluate accuracy between bound and unbound conditions but fail to quantify the error itself), the results presented in this paper suggest that the localisation performance metric, instead of the swap error proportion, may be more sensitive to preclinical decline. The localisation performance metric measures the distance between the centre of the target object once placed in its remembered location and its true (original) location in the memory array *after* the correct fractal has been identified. Therefore, it could be argued that this metric indicates the resolution or quality of recall of the object's identity *bound* to its exact location – with greater error indicating less memory precision. Importantly, presymptomatic deficits were observed in both the localisation error and swap proportion metrics in Liang and colleagues report (Liang et al., 2016) and the findings presented here indicate that localisation performance could represent the resolution or accuracy of relational binding more sensitively than the swap error proportion – at least with regards to tracking presymptomatic decline.

In line with this proposal, in the same way that the ‘shape-only’ condition in a change-detection shape-colour conjunctive binding task, accounts for the ‘unbound’ condition; the identification performance metric here, may represent the ‘unbound’ condition. While a significantly faster rate of decline in identification performance was also observed in symptomatic carriers in addition to localisation performance, the deficit in late PMCs was specific to localisation performance. Furthermore, group differences between late PMCs and controls were reduced when comparing rates of change in the ‘nearest item control’ metric (see Supplementary Materials, section 2.2). This suggests that some of the imprecision in remembering the object's identity and location, may be explained by a tendency to mislocalise the fractal to the location of another fractal (regardless of whether it was the target) and that the swap error proportion – being a binary metric – may not have been sensitive enough to detect this subtle change.

The focus on localisation performance as a measure of relational binding accuracy is in line with more recent views of working memory models, specifically resource models, in which recall declines gradually and continuously with increasing number of items as resources are flexibly distributed (Bays and Husain, 2008; Wilken and Ma, 2004). Importantly, localisation precision decreased (the degree of error increased) when three fractals were presented in comparison to one. In this regard, it may be relevant to note a recent study by Weston and colleagues (Weston et al., 2020) showing higher mean diffusivity for PMCs than controls in the precuneus – a region important for mental or visuo-spatial imagery and closely related to working memory (Baddeley, 2003). This emphasizes that quantifying precision in space (e.g. through a continuous measure like localisation performance), may be best suited to detect subtle cognitive changes in PMCs. Additionally, as these effects were predominant in long delay conditions, the impairment observed in late PMCs over time, may be related to a difficulty in maintenance processes.

We speculate that our longitudinal findings may be explained by a ‘unified account of hippocampal forgetting across short and long timescales’, proposed recently by Sadeh and Pertzov (2020) according to which the similarities between short (interval of a few seconds between study and test, e.g. STM or working memory paradigms) and long timescales (study-test intervals of several minutes to days/months) suggests that a single hippocampus-based mechanism underlies memory in both timescales. This contrasts the once prevailing view that the hippocampus (proposed to be one of the earliest regions affected by AD pathology by some (Chan et al., 2016; Fox et al., 1996; Liang et al., 2017)) was exclusively involved in memory and forgetting over long timescales. We propose that a process similar to accelerated forgetting may provide an explanation for the deficits observed and that the passage of time may be a source of forgetting in PMCs. Accelerated forgetting refers to a long-term memory process whereby new material appears to be encoded and retained normally over periods of up to 30 min but is then forgotten at an abnormally rapid rate over the following hours to weeks (Weston et al., 2018). Analogously, our results suggest that the precision of localisation performance in VSTM for late PMCs, declined at an accelerated rate compared to controls. While the exact mechanism for this process is poorly understood, the hippocampus has been implicated in the formation and retention of memories (Squire, 2009). Notably, previous reports from our centre have shown strong correlations between localisation performance and hippocampal volume (once adjusted for age, sex and total intracranial volume). Therefore, there may be processes specific to the hippocampus which underly this ‘accelerated forgetting’ phenomenon across time-scales – though other reports have shown that atrophy in the entorhinal cortex precedes that in the hippocampus (Braak and Braak, 1991; Liang et al., 2017) and these topics are still under debate in the field of AD.

### 4.3 Integrating VSTM results with previous literature

Unlike reports from our centre by Liang and colleagues showing a higher proportion of swap errors in PMCs (Liang et al., 2016), cross-sectional deficits presented here were only observed in symptomatic carriers. In addition, there was no difference in the rate of change of swap errors for any of the patient groups compared to controls. While the separation of PMCs into ‘late’ and ‘early’ is novel, PMCs as a whole did not show a greater proportion of swap errors either (see Supplementary Materials, Fig-e5).

A number of reasons may explain these differences. Firstly, differences in the characteristics of the PMCs sample in comparison to Liang and colleagues' report (Liang et al., 2016) may have influenced results. The inclusion of more PMC participants (23 in our study *vs* 12 in Liang and colleagues' and 6 additional mutations – 5 *PSEN1* and 1 *APP*) meant that they were on average further from expected onset and had a broader range of EYO in comparison to Liang and colleagues' report (mean EYO = 9.5 (SD 5.0) *vs* 8.5 (3.8)). This may have resulted in performance differences given that disease progression varies between genes (with *PSEN1* mutation carriers more frequently presenting with non-amnestic cognitive symptoms than *APP* mutation carriers (Ryan et al., 2016; Scahill et al., 2013)) and even between mutations within the same gene (Pavisic et al., 2020b). Furthermore, late PMCs in our study had lower anxiety scores compared to controls (in both N = 99 and N = 48 samples) and this was not the case for Liang and colleagues’ PMC group (in which patients and controls had similar anxiety scores comparable to our control group (Liang et al., 2016)). While high anxiety levels have shown to negatively impact cognition (Okon-Singer et al., 2015) and visual working memory specifically (e.g. (Spalding et al., 2020)), implications of low anxiety scores on cognition are complex and it is difficult to establish whether or not this could have carried some advantage for late PMCs performance especially in light of the reduced insight that may be observed sometimes in presymptomatic stages of FAD. Lastly, as a relatively accurate localisation is required for a response to count as a swap, swap errors may have been underrepresented in our study (in both symptomatic and presymptomatic carriers) especially in light of the localisation error finding. The non-significant interaction between the rate of swap error proportion and delay in our longitudinal analysis was also surprising, yet the worsening localisation particularly for longer delays may have veiled this interaction too.

A critical advantage of longitudinal studies over cross-sectional investigations is the ability to assess when changes occur. Recently, a proposal emerged suggesting that AD progressed in two stages: a sub-hippocampal phase characterised by impairments in context-free memory function such as those assessed by recognition tasks, followed by a hippocampal stage when impairments in context-rich memory functions (such as ‘associative memory’) are observed and which corresponds clinically to the stage at which cognitive impairment is evident (Parra, 2017). In this respect, it is relevant to note that the longitudinal performance of PMCs was significantly worse than controls both in the ‘What was where?” task (arguably a context-rich memory function) and in the RMT for words and that changes in the localisation metric of the “What was where?” task were observed at least as early as changes in the RMT for words task. Decline in recognition memory tests have typically been associated with AD (e.g. (Diesfeldt, 1990)) and while most sensitivity has been described in symptomatic AD, some reports suggest recognition discriminability for amnestic mild cognitive impairment patients with biomarker evidence of prodromal AD (Goldstein et al., 2019). However, the relative sensitivities and multicomponent nature of each test certainly affect findings. For example, certain brain areas which are active during the episodic retrieval of recognition tests (e.g. the right anterior prefrontal cortex (Rugg et al., 1998) or the entorhinal cortex (Weston et al., 2016)), might also overlap with the neural correlates of binding (frontal-parietal-MTL network for conjunctive binding and parietal-occipital-temporal networks for relational binding) (Jonin et al., 2019; Parra et al., 2015). Longitudinal imaging studies including physiological measures such as functional MRI, in preclinical AD populations like presymptomatic FAD, are therefore needed to further establish which regions of the brain show significant deficits in preclinical AD in comparison to controls and in which order.

### 4.4 Limitations

The current study has several limitations. Firstly, despite the increased sample size in comparison to the previous cross-sectional study (23 presymptomatic carriers in this study *vs* 12 presymptomatic carriers in (Liang et al., 2016)), this remains relatively small due to the low prevalence of FAD. Secondly, disease progression is complex and not well characterised in the literature, especially in FAD (Canevelli et al., 2014; Pavisic et al., 2020b; Ryman et al., 2014; Shea et al., 2016). As our study included mutation carriers from pedigrees with different *PSEN1* and *APP* mutations, it is possible that by considering all FAD carriers together, the heterogeneity in the progression of the disease between genes and mutations may have affected our results. However, creating mutation-based subgroups would not have been possible due to issues around validity of modelling such small groups. Furthermore, ‘late’ PMCs were a heterogenous group in that individuals' EYO spanned within 8.5 years before expected onset; mean = −5.8 (SD 1.8) years and these estimations are imprecise given the within-family variation in age at onset (Ryman et al., 2014). The exploratory analysis done separating PMCs by the median split has its limitations too, as some participants may lie close to each other in EYO but be classified into different groups. Yet, the investigation of VSTM performance and EYO in a continuous scale was presented as a complementary approach, partly for these reasons. Thirdly, the qualitative observation of VSTM performance in ‘converters’ showed that for all VSTM metrics, performance did not follow a unique pattern once participants transitioned into a symptomatic stage (for some participants, scores worsened while for others they remained stable). Reporting this substantial variability possibly resulting from the 100 trials completed by participants at every visit in addition to the limitations previously mentioned, is important as it raises novel considerations of the use of such tasks at an individual level. Lastly, our findings may also be explained by the attention and frontal/executive demands of this task (with the localisation measure being particularly sensitive due to its continuous nature), rather than the visuo-spatial or memory aspects *per se* as well as differences in life-course factors (e.g. socio-economic status and occupation type), some of which have been shown to impact the onset and rate of cognitive decline in individuals with certain FAD mutations (e.g. (Aguirre-Acevedo et al., 2016)).

Taken together, the findings presented in this paper have important implications for the usefulness and reliability of this task especially as certain deficits in PMCs (e.g. swap errors) were not replicated. Notably, a recent FAD study by Norton and colleagues (Norton et al., 2020), found that the condition requiring conjunctive binding of colour and shape was not preferentially linked to tau (measured by positron emission tomography – PET) but rather that the non-binding “shape only” condition showed a stronger relationship. This emphasizes that much remains to be understood about VSTM binding and that group studies comparing conjunctive and relational binding over time, will provide critical data on which processes are affected, at what stage and in which order.

Longitudinally, the observation that deficits were seen in the late PMCs but not in early PMCs, raises important questions as to when the “What was where?” task – or relational binding as a cognitive function – may be sensitive to tracking preclinical decline in AD. In this regard it is worth noting that in the analysis of EYO as a continuous measure, deficits were seen up to 6 years before estimated age at symptom onset (the mean EYO of late PMCs). Future studies with greater sample sizes and a broader range of EYO, should investigate questions around usefulness and reliability further.

## 4.5 Conclusions

To the best of our knowledge, this is the first longitudinal investigation on VSTM function in a preclinical sample like FAD over a period of many years. Our findings highlight that evaluating the *degree* of error on a continuous scale may be a sensitive measure of longitudinal decline in the presymptomatic stages of FAD. Analogous to the accelerated-forgetting hypothesis, we speculate a similar phenomenon may explain VSTM deficits, whereby the ability to spatially remember and retain a memory representation is forgotten with time at an ‘accelerated rate’ in presymptomatic FAD compared to controls.

More broadly, these results merit further exploration particularly in light of the similarities between sporadic and familial AD and the importance of identifying and tracking individuals at-risk of developing AD as early as possible for intervention trials. Future longitudinal studies, which ideally administer conjunctive and relational binding tasks to the same sample, may wish to investigate correlation of performance with functional outcomes, hippocampal volume or amyloid beta deposition, in order to further validate its use for screening and monitoring purposes in FAD and other preclinical AD populations more broadly.

## Declaration of interests

I.M. Pavisic; J.M. Nicholas; Y. Pertzov; A. O'Connor; Y. Liang; J. D. Collins; K. Lu; P.S.J. Weston; N.S. Ryan; M. Husain and S. Crutch report no disclosures relevant to this manuscript. N.C. Fox has provided consultancy for Biogen, Ionis and Roche and serves on a Data Safety Monitoring Committee for Biogen.

## Availability of data and material

The datasets used and/or analysed during the current study are available from the corresponding author on reasonable request.

## Appendix A Supplementary data

This includes further details on the participants of this study; statistical analysis; baseline results for the longitudinal sample; findings on the nearest item control metric; five additional figures and one additional table.
