# Supplementary material for: Visual short-term memory impairments in presymptomatic familial Alzheimer's disease: A longitudinal observational study
Source: Neuropsychologia. 2021 Nov 12;162:108028. doi: 10.1016/j.neuropsychologia.2021.108028 (PMC8589962; doi:10.1016/j.neuropsychologia.2021.108028)
Supplement: Multimedia component 1 [file mmc1.pdf]

# SUPPLEMENTARY MATERIAL

## Visual short-term memory impairments in presymptomatic familial Alzheimer's disease: A longitudinal observational study – Pavisic et al

Here we provide supplementary information on methods and results including: details on the participants included in this study; statistical analysis; baseline results for the longitudinal sample and findings on the nearest item control (NIC) metric.

## 1. Supplementary Methods

### *1.1. Participants included*

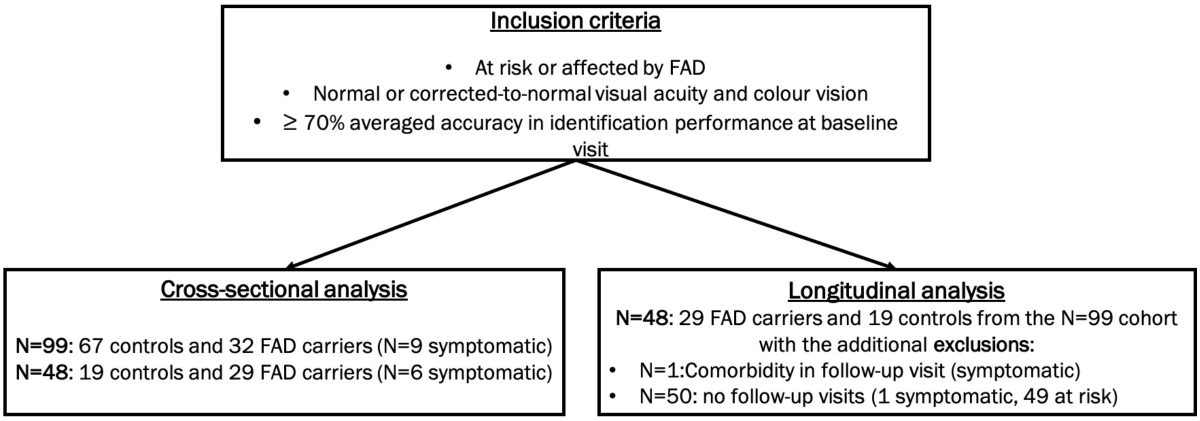


**Figure-e1. Participants included in the analyses.**

Differences between our N=99 and Liang et al., 2016 study

**Additions:**

- N=14 - first visit was after the original study (13 at-risk, 1 symptomatic) – with these individuals, 5 additional *PSEN1* mutations and 1 additional *APP* mutation were added in comparison to Liang and colleagues report.
- N=4 - mutation carrier status has become available since the original study (all at-risk)

**Exclusions:**

- N=1 - missing neuropsychology (at-risk)

### *1.2. Additional information on statistical methods*

Due to a skewed distribution the absolute localisation error and NIC were log transformed and proportion of swap errors was square root transformed before analysis. Longitudinal change in object identity was analysed using a mixed effects logistic regression model and analysis of the other VSTM outcomes used a linear mixed effects model. In the **longitudinal analysis of VSTM with groups by symptom status and categories of EYO:** for each outcome, rates of change were compared between groups (symptomatic carriers, early PMCs, late PMCs and controls) by including group at the baseline assessment and an interaction between group at the baseline assessment and follow-up length as predictors in each model. Models were adjusted for delay, block, number of items (where relevant), sex, age at baseline, and NART at baseline. The linear mixed effects models also included separate residual error terms for symptomatic mutation carriers, PMCs, and controls to allow for heteroscedasticity. Models included a random slope and intercept to allow clustering by participant. The models for localisation and NIC error additionally included a random effect of visit, nested in participant. Random effects were assumed to follow a normal distribution. For example, the linear mixed model for localisation was specified as:

$$\begin{aligned} y_{ijk} = \beta_0 + (\beta_1 + b_i)t_{ij} + \beta_2 ePMC_i + \beta_3 lPMC_i + \beta_4 symp_i + \beta_4 ePMC_i * t_{ij} + \beta_5 lPMC_i * t_{ij} \\ + \beta_6 symp * t_{ij} + \beta_7 item_{ijk} + \beta_8 delay_{ijk} + \beta_9 block_{ijk} + \beta_{10} age_i + \beta_{11} sex_i \\ + \beta_{12} NART_i + \mu_i + \omega_{ij} + \varepsilon_{ijk} \end{aligned}$$

Where,

$y_{ijk}$ is the log localisation error for the kth item at visit j for the ith individual

$t_{ij}$ is the time from baseline to visit j for the ith individual

$ePMC_i$, $lPMC_i$ and $symp_i$ are binary indicator variables taking the value 1 if the ith individual is in the early PMC, late PMC or symptomatic group respectively and 0 otherwise

$item_{ijk}$ is a binary indicator variable taking the value 1 if the kth item at visit j for the ith individual had 3 fractals and 0 otherwise

$delay_{ijk}$ is a binary indicator variable taking the value 1 if the kth item at visit j for the ith individual had a delay of 4s and 0 otherwise

$block_{ijk}$ is a binary indicator variable taking the value 1 if the kth item at visit j for the ith individual was in block 2 and 0 otherwise

$age_i$ is the age at baseline in years for the ith individual

$sex_i$ is a binary indicator variable taking the value 1 if the ith individual is female and 0 otherwise

$NART_i$ is the value of NART at baseline for the ith individual

$b_i$ , $\mu_i, \omega_{ij}$, are random effects for slope, intercept and visit

$\varepsilon_{ijk}$ is the residual error

A joint Wald test of the three interaction coefficients between group and time (i.e. coefficients for symp*t, ePMC*t, lPMC*t) was used to examine whether the rate of change differed between groups. Where differences were found, further Wald tests of each interaction coefficient were conducted to examine each group's difference from controls in the rate of change. To examine whether the mean performance differed between groups a joint Wald test was done for the six coefficients involving group (i.e. for symp, ePMC, lPMC, symp*t, ePMC*t, lPMC*t). Where differences were found, further Wald tests were done on each pair of coefficients to examine the difference for that group versus controls (e.g. test of symp and symp*t for the symptomatic group).

To examine whether group differences in rates of change in performance varied by delay, block and number of items, a two-way interaction between condition and group, two-way interaction between condition and time, and three-way interaction between group, condition and time were added to the model. A Wald test of the 3 three-way interaction terms was used to examine whether this condition influenced the differences between groups in rate of change. For example, a test for interaction on number of items would test the terms associated with symp*t*item, ePMC*t*item, and lPMC*t*item. Where interactions with group were found, further Wald tests were done for each coefficient to examine whether there was evidence of an interaction for that group (e.g. test symp*t*item for the symptomatic group). A Wald test of the 6 interaction terms between condition and group was used to examine whether this condition influence the differences by group. For example, a test for interaction on number of items would test the terms associated with symp*item, ePMC*item, lPMC*item, symp*t*item, ePMC*t*item, and lPMC*t*item. Where interactions with group were found, further Wald tests were done to examine whether there was evidence of an interaction for that group (e.g. test symp*item and symp*t*item for the symptomatic group). The resulting models were used to estimate the marginal mean score in each group and differences in mean score between each group and controls for visits up to 3 years after baseline, by condition where interactions were found.

In the **longitudinal analysis of VSTM using EYO as a continuous measure:** for each outcome, performance was compared between gene carriers and controls by including as predictors age at visit, carrier status, and in the carriers, years to expected onset and years to expected onset squared. Separate random intercept terms were included for controls and for mutation carriers to allow for clustering by participant. A random slope for time to expected onset was also included where this significantly improved the model fit based on a likelihood ratio test. For example, the linear mixed model for localisation was specified as:

$$y_{ijk} = \beta_0 + (\beta_1 + b_i)MC_i * EYO_{ij} + \beta_2 MC_i * EYO_{ij}^2 + \beta_4 MC_i + \beta_5 age_{ij} + \beta_6 item_{ijk} + \beta_7 delay_{ijk} + \beta_8 block_{ijk} + \beta_9 sex_i + \beta_{10} NART_i + \mu_i + \omega_{ij} + \varepsilon_{ijk}$$

Where,

$y_{ijk}$ is the log localisation error for the kth item at visit j for the ith individual

$age_{ij}$ is the age in years at visit j for the ith individual

$EYO_{ij}$ is the expected years to onset at visit j for the ith individual (mutation carriers only)

$MC_i,$ is a binary indicator variable taking the value 1 if the ith individual is a mutation carrier and 0 otherwise

$item_{ijk}$ is a binary indicator variable taking the value 1 if the kth item at visit j for the ith individual had 3 fractals and 0 otherwise

$delay_{ijk}$ is a binary indicator variable taking the value 1 if the kth item at visit j for the ith individual had a delay of 4s and 0 otherwise

$block_{ijk}$ is a binary indicator variable taking the value 1 if the kth item at visit j for the ith individual was in block 2 and 0 otherwise

$sex_i$ is a binary indicator variable taking the value 1 if the ith individual is female and 0 otherwise

$NART_i$ is the value of NART at baseline for the ith individual

$b_i$ , $\mu_i, \omega_{ij}$ are random effects for slope, intercept and visit

$\varepsilon_{ijk}$ is the residual error

A joint Wald test of the two coefficients for EYO (i.e. for MC*EYO and MC*EYO$^2$) was used to examine whether there was an association with EYO in the mutation carrier group and a Wald test for the three MC coefficients (i.e. for MC, MC*EYO and MC*EYO$^2$) was used to examine whether there was evidence of a difference in score between carriers and non-carriers. To examine whether the association with years to expected onset varied by delay, block and number of items, interactions were added between the condition and carrier status, condition and years to expected onset and condition and years to expected onset squared. A joint Wald test of the two interaction coefficients for EYO was used to examine whether there was evidence of a difference in the association with EYO by condition (e.g. for item this would be a test of item*MC*EYO and item*MC*EYO$^2$) and joint Wald test of the carrier status interaction and two interaction coefficients for EYO was used to examine whether there was evidence that difference between mutation carriers and controls differed by condition (i.e. for item this would be a test of item*MC, item*MC*EYO and item*MC*EYO$^2$). The resulting models were used to estimate the marginal mean score in each group and differences in mean score between each group and controls by expected years to onset (and by condition where interactions were found).

To consider whether differences between groups may be due to motor function difficulties rather than deficits in recall, we conducted a post-hoc analysis using a measure of motor function quantified as the difference in degrees between the participant selected location within the stimulus and the centre of the stimulus (for correctly identified fractals only). Due to a skewed distribution this was log transformed before analysis. In this **longitudinal analysis of motor function with groups by symptom status and categories of EYO:** a linear mixed model was used to compare motor function and rates of change between groups (symptomatic carriers, early PMCs, late PMCs and controls) by including group at the baseline assessment and an interaction between group at the baseline assessment and follow-up length as predictors in the model. The model was also adjusted for delay, block, number of items, sex, age at baseline, and NART at baseline. The models included a random slope and intercept to allow clustering by participant, a random effect of visit nested in participant and separate residual error terms for symptomatic mutation carriers, PMCs, and controls to allow for heteroscedasticity. The same Wald tests as for other VTSM analysis were used to examine whether there was evidence of a difference between the groups in motor function or rate of change in motor function. No tests were conducted for interactions by number of items, delay or block given the focus on motor function.

In the **longitudinal analysis of neuropsychology with groups by symptom status and EYO:** for each outcome, rates of change were compared between group (symptomatic carriers, early PMCs, late PMCs and controls) by including group at the baseline assessment and an interaction between group at the baseline assessment and follow-up length as predictors in each model. Due to skewed data a cubic transform was used for BPVS and inverse for Stroop time prior to analysis. To allow for clustering at a participant level random effects were included, with separate random effects by carrier status included where these additional terms improved model fit. The random effects included in the linear regression models were: a random intercept for GNT, NART and Stroop; a random intercept and slope for BPVS; and a random slope and intercept for carriers and a separate random intercept for controls for performance IQ, verbal IQ and arithmetic. The regression models for RMT words, RMT faces, VOSP, digit span forwards and digit span backwards included separate random intercepts for carriers and controls. All the mixed effects linear regression models had separate residual error terms for symptomatic carriers, PMCs and controls to allow for heteroscedasticity. As above for the VTSM metrics a joint Wald test of the three interaction coefficients between group and time (i.e. for symp*t, ePMC*t, lPMC*t) was used to examine whether the rate of change differed between groups. Where differences were found, further Wald tests of each interaction coefficient were conducted to examine each group's difference from control in the rate of change. To examine whether the mean performance differed between groups a joint Wald test was done for the six coefficients involving group (i.e. for symp, ePMC, lPMC, symp*t, ePMC*t, lPMC*t). Where differences were found, further Wald tests were done on each pair of coefficients to examine the difference for that group versus controls (e.g. test of coefficients for symp and symp*t for the symptomatic group).

## 2. Supplementary Results

### *2.1. Baseline results for subgroups of participants included in the longitudinal analysis (N=48)*

#### *2.1.1. Demographics and traditional neuropsychology*

Nineteen controls and 29 carriers with longitudinal data on the VSTM binding task were included. Early PMCs were 12.6 years before EYO and on average younger than the control group ($p$=0.041) and. Late PMCs were on average 5.8 years before EYO. Baseline anxiety and depression scores were slightly lower for the late PMCs compared to controls (anxiety: $p$=0.023, depression: $p$=0.049, Table e1). As expected, symptomatic carriers were older ($p$=0.029), had lower MMSE ($p$=0.002) and were on average 2.7 years after expected onset at the baseline visit (Table e1). Once again, global CDR scores were consistent with the early stages of symptomatic AD (mean=0.6 (SD 0.2), range= 0.5 – 1, Table e1).

Compared to controls, there was evidence that the early PMC group had higher scores for backwards digit span ($p$=0.049) and late PMCs had lower values for performance IQ ($p$=0.005, Table e1). Symptomatic individuals were on average, significantly worse than controls on arithmetic ($p$=0.018), RMT for words ($p$<0.001) and Stroop ($p$=0.019) and tended to have lower performance IQ scores ($p$=0.076, Table e1).

#### *2.1.2. VSTM performance*

Symptomatic carriers had 41.0 [16.5, 38.3] % lower odds of correctly identifying the target (OR=0.59, $p$=0.003), 53.0 [18.1, 98.3] % greater localisation error ($p$=0.002) and made a greater proportion of swap errors (difference in √swap error proportion at baseline= 0.208 [0.136, 0.280], $p$<0.001) in comparison to controls. There was also a trend for symptomatic participants to have greater error at localising the nearest fractal (15.8 [-1.4, 35.9] % greater error, $p$=0.080) (Table e1). Late PMCs were significantly worse than controls at localising the nearest fractal at baseline (16.9 [3.6, 31.9] % greater error, $p$=0.015) but no significant differences were seen from controls for the other measures (Table e1). Early PMCs had similar performance to controls on all measures (Table e1, Fig.e-2).

A significant interaction of group with block was observed for the identification measure ($p$=0.020) with symptomatic carriers showing a much larger difference from controls in block 2 (61.8 [39.8, 75.7] % lower odds of correct identification, $p$<0.001) than in block 1 (14.1 [-31.8, 44.5] % lower odds of correct identification, $p$=0.479). A trend towards an interaction of group with increasing memory load was seen for the localisation measure ($p$=0.067), whereby symptomatic carriers showed greater differences from controls in the 3-item *vs* the 1-item condition (Table e1). There were no significant interactions with group for the proportion of swap errors (delay: $p$=0.117; block: $p$=0.273). However, following Liang and colleagues (Liang et al. 2016) we investigated performance in the 4s delay, block 1 condition. A significantly greater swap error proportion from controls was seen only for symptomatic

carriers (difference in √swap error proportion at baseline=0.196 [0.054, 0.339], $p$=0.008, Fig-e2; also see Table e1 for unadjusted group means).

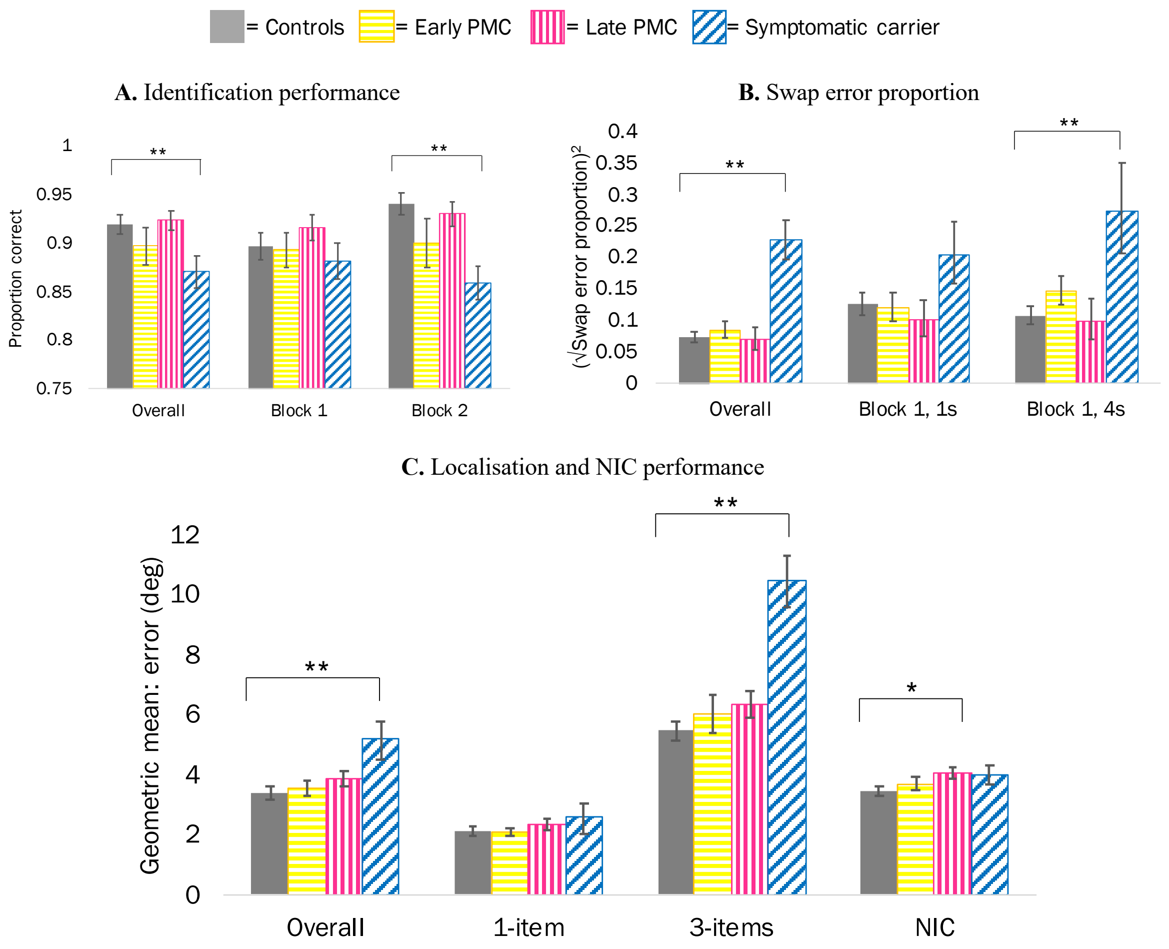


**Figure-e2. Cross-sectional adjusted mean performance by group for the longitudinal sample (from model adjusted for age, sex and NART at baseline). A.** Identification performance (across all conditions); **B.** Swap error proportion across all conditions and in the 4s delay, block 1 condition. **C.** Localisation and nearest item control (NIC) performance by item-number. Note that the NIC is by definition calculated with 3-items only. Error bars show +/- standard error of the mean. PMC=presymptomatic mutation carrier. *= significant at $p$<0.05; **: significant at $p$<0.01.

**Table e1.** Baseline demographics, neuropsychology and VSTM performance by group for participants in the longitudinal analysis (N=48).

| | Controls (N=19) | Early PMCs (N=12) | Late PMCs (N=11) | Symptomatic carriers (N=6) |
|---|---|---|---|---|
| **Demographics** | | | | |
| Sex: N (%) Male | 9 (47.4) | 3 (25.0) | 7 (63.6) | 4 (66.7) |
| Age (yrs) | 41.2 (9.4) | **34.8 (6.4)*** | 37.0 (5.0) | **50.0 (11.8)*** |
| EYO (yrs) | NA | -12.6 (4.7) | -5.8 (1.8) | 2.7 (4.3) |
| AYO (yrs) | NA | NA | NA | 2.0 (2.4) |
| Education (yrs) | 14.2 (2.7) | 14.3 (2.5) | 13.3 (2.5) | 15.0 (3.0) |
| MMSE | 29.6 (0.7) | 29.3 (0.9) | 29.5 (0.8) | **25.3 (3.7)**** |
| CDR global | 0.0 (0.0) | 0.0 (0.0) | 0.0 (0.0) | **0.6 (0.2)**** |
| Anxiety | 6.9 (3.9) | 7.8 (4.8) | **3.9 (3.9)*** | 6.0 (4.7) |
| Depression | 3.5 (3.4) | 3.1 (4.2) | **1.3 (1.6)*** | 1.7 (1.8) |
| **Neuropsychology** | | | | |
| Performance IQ | 113.5 (7.5) | 106.5 (15.0) | **101.4 (10.1)**** | 104.3 (12.1) |
| Verbal IQ | 106.7 (15.9) | 96.9 (14.6) | 95.4 (13.5) | 102.7 (20.3) |
| Arithmetic total/24 | 17.2 (5.4) | 13.9 (5.0) | 14.3 (4.6) | **11.2 (6.3)*** |
| RMT faces | 42.5 (4.2) | 41.1 (4.2) | 43.8 (4.5) | 41.2 (4.3) |
| RMT words | 47.6 (2.8) | 48.7 (2.2) | 46.5 (2.8) | **38.0 (4.0)**** |
| Digit span forwards/8 | 6.8 (1.2) | 6.8 (1.0) | 7.4 (1.1) | 6.3 (1.5) |
| Digit span backwards/7 | 4.8 (1.1) | **5.7 (1.3)*** | 5.4 (1.1) | 4.8 (1.6) |
| BPVS | 140.9 (9.5) | 135.0 (14.4) | 139.8 (10.1) | 140.5 (11.1) |
| GNT/30 | 20.3 (4.3) | 17.8 (5.8) | 19.2 (5.4) | 22.0 (4.6) |
| NART/50 | 28.6 (9.0) | 24.1 (8.6) | 27.7 (10.7) | 29.7 (12.1) |
| VOSP OD /20 | 17.9 (2.1) | 17.8 (1.8) | 18.3 (1.3) | 18.2 (1.2) |
| Stroop (s) | 49.8 (12.8) | 47.6 (8.7) | 52.6 (14.1) | **73.3 (25.8)*** |
| **VSTM performance** | | | | |
| **Identification (% correct)** | | | | |
| Overall | 91.3 (4.6) | 89.9 (6.3) | 92.0 (3.9) | **83.7 (5.3)**** |
| Block 1 | 88.9 (6.5) | 89.8 (5.9 | 91.3 (4.3) | 85.0 (5.5) |
| Block 2 | 93.6 (5.4) | 90.2 (8.8) | 92.7 (5.3) | **82.3 (6.1)**** |
| **Localisation error (deg)** | | | | |
| Overall | 5.1 (1.1) | 5.8 (2.0) | 5.7 (1.5) | **9.6 (1.6)**** |
| 1-item, (all delays) # | 2.3 (0.7) | 2.3 (0.3) | 2.5 (0.8) | 3.1 (1.7) |
| 3-items, (all delays) # | 5.9 (1.4) | 6.8 (2.5) | 6.6 (1.8) | **11.6 (1.8)**** |
| **Nearest item control (deg)** | | | | |
| Overall: all delays | 3.3 (0.6) | 3.5 (0.8) | **3.7 (0.6)*** | 4.0 (0.8) |
| **Swap error proportion (%)** | | | | |
| Overall | 9.4 (3.1) | 12.2 (4.6) | 10.2 (5.9) | **25.8 (7.7)**** |
| Block 1, 1s delay | 12.1 (6.3) | 12.9 (9.0) | 9.9 (5.0) | **24.2 (14.9)*** |
| Block 1, 4s delay | 13.9 (5.9) | 18.3 (9.4) | 15.0 (10.8) | **30.1 (18.5)**** |

Unadjusted mean values are given with SD unless otherwise stated. SD = standard deviation; NA= not applicable; PMC= presymptomatic mutation carrier; EYO=years to/from predicted symptom onset (a negative value indicates a younger age than their estimated age at symptom onset); AYO=actual years to/from onset (positive values indicate years post onset; Anxiety and depression measures scores were taken from the HADS= hospital anxiety and depression scale; IQ=intelligence quotient; Digit spans forwards and backwards are taken from the WMS-R= Wechsler Memory Scale; RMT=recognition memory test; GNT=graded naming test. #localisation measures are separated by item-number to allow for comparison with NIC findings. Neuropsychology data were available at baseline for: 47 participants for performance IQ, verbal IQ and Stroop; for all 48 participants for the remaining tests. Bold = significant; *: the difference between the patient group and controls for that variable was significant at $p<0.05$; **: the difference between the patient group and controls for that variable was significant at $p<0.01$.

### 2.2. Nearest item control (NIC) results

#### 2.2.1. Cross-sectional results (N=99)

The adjusted group mean error was 3.6 [95 % CI 3.4, 3.8] deg for control, 3.6 [3.2, 4.0] deg for early PMCs, 4.0 [3.6, 4.4] deg for late PMCs and 4.2 [3.7, 4.9] deg for symptomatic carriers. Symptomatic carriers had significantly greater error in the NIC metric compared to controls (17.5 [95% CI 1.4, 36.2] % greater error, $p$=0.032). While there was a trend for higher NIC error in the late PMC group, this did not reach statistical significance (10.5 [-1.4, 23.9] % greater error $p$=0.085). NIC error was similar between early PMCs and controls (-0.2 [-12.9, 14.5] % difference, $p$=0.980). There was no significant interaction between group and delay ($p$=0.236) or block ($p$=0.454).

For comparison, in the 3-item condition the adjusted mean localisation error (without NIC) was 6.0 [5.6, 6.5] deg for controls, 5.9 [4.8, 7.3] deg for early PMCs, 6.3 [5.5, 7.2] deg for late PMCs, and 10.0 [8.6, 11.7] deg for symptomatic carriers. Symptomatic carriers had significantly greater localisation error compared to controls in the 3-item condition (67.2 [95% CI 41.4, 97.5] % greater localisation error, $p$<0.001). The was little evidence that either PMC group had higher localisation error than controls (late PMCs: 5.1 [-10.4, 23.4] % difference, $p$=0.534; early PMCs: -0.9 [-21.0, 24.3] % difference $p$=0.938).

The finding of much smaller difference in localisation between symptomatic carriers and controls after NIC suggests that some of the greater localisation error in this group at baseline may be accounted for by a tendency to mislocalise the fractal to the location of the nearest fractal (regardless of whether it was the target).

#### 2.2.2. Longitudinal results (N=48)

##### 2.2.2.1. Rates of change

NIC performance of controls generally stayed the same throughout the course of the study (0.6 [-1.6, 2.7] %, $p$=0.607). There was a trend for greater error for early PMCs (2.3 [-0.1, 4.8] %, $p$=0.057) and a significantly poorer performance for late PMCs (2.7 [0.1, 5.5] %, $p$=0.045) and symptomatic carriers (7.6 [2.3, 13.1] %, $p$=0.004).

PMCs did not show a significant difference in the rate of NIC error per year compared to controls (early PMCs=1.8 [-1.4, 5.1] % greater error per year, $p$=0.281; late PMCs= 2.2 [-1.2, 5.7] % greater error per year, $p$=0.215). Symptomatic carriers on the other hand, had faster increase in NIC localisation error compared to controls (7.0 [1.3, 12.9] % greater error per year, $p$=0.015). This suggests that the increase in localisation error observed for the symptomatic group (6.5 [-0.4, 13.9] % greater error per year) was not accounted for by a tendency to mislocalise the fractal to the location of the nearest fractal (regardless of whether it was the target).

While no significant interactions of group rate of change emerged for delay (*p*=0.364) or block (*p*=0.986), delay conditions were evaluated separately given the findings for localisation error. Symptomatic carriers showed a significantly faster increase in NIC error compared to controls in the 4s delay condition (9.1 [1.8, 17.0] % greater error per year, *p*=0.013) and a trend in the same direction in 1s delay condition (5.1 [-1.7, 12.3] % greater error per year, *p*=0.114). There was a suggestion that late PMC group may have faster increase in NIC error compared to controls specific to the 4s delay condition (3.9 [-0.2, 8.2] % greater error per year, *p*=0.064) as no difference was seen for the 1s delay condition (0.6 [-3.4, 4.7] % greater error per year, *p*=0.778). There was a weak trend for the early PMC group to have faster increase in NIC error than controls in the 4s condition (3.1 [-0.7, 7.0] % greater error per year, *p*=0.112) but not 1s condition (0.5 [-3.2, 4.3] % greater error per year, *p*=0.804). A comparison between NIC (3.9 [-0.2, 8.2] % greater error per year) and localisation (6.9 [1.8, 12.2] % greater error per year) error differences in late PMCs compared to controls in the 4s condition suggested that some, but not all, of the difference from controls in localisation error could be accounted by a tendency to mislocalise the fractal to the location of the nearest fractal (regardless of whether it was the target). However, as some of the difference from controls remained with NIC, this indicated that part of the increase in localisation error was specific to the target distance rather than solely an effect of mislocalising the fractal.

#### *2.2.2.2. Relationship with proximity to symptom onset*

Considering all FAD (presymptomatic and symptomatic) carriers, there was a trend towards greater NIC error with EYO (*p*=0.068). Although there was no significant interaction with delay (*p*=0.082), delay conditions were examined separately for comparison to localisation error performance. In the 4s condition, there was a significant association with EYO (*p*=0.036, Fig.e4A), with a difference in NIC error between FAD carriers and controls observed 6 years prior to EYO (difference=13.0 [0.6, 27.0] %, *p*=0.023). There was also a significant association between worsening NIC performance and AYO (*p*=0.002, Fig-e4B).

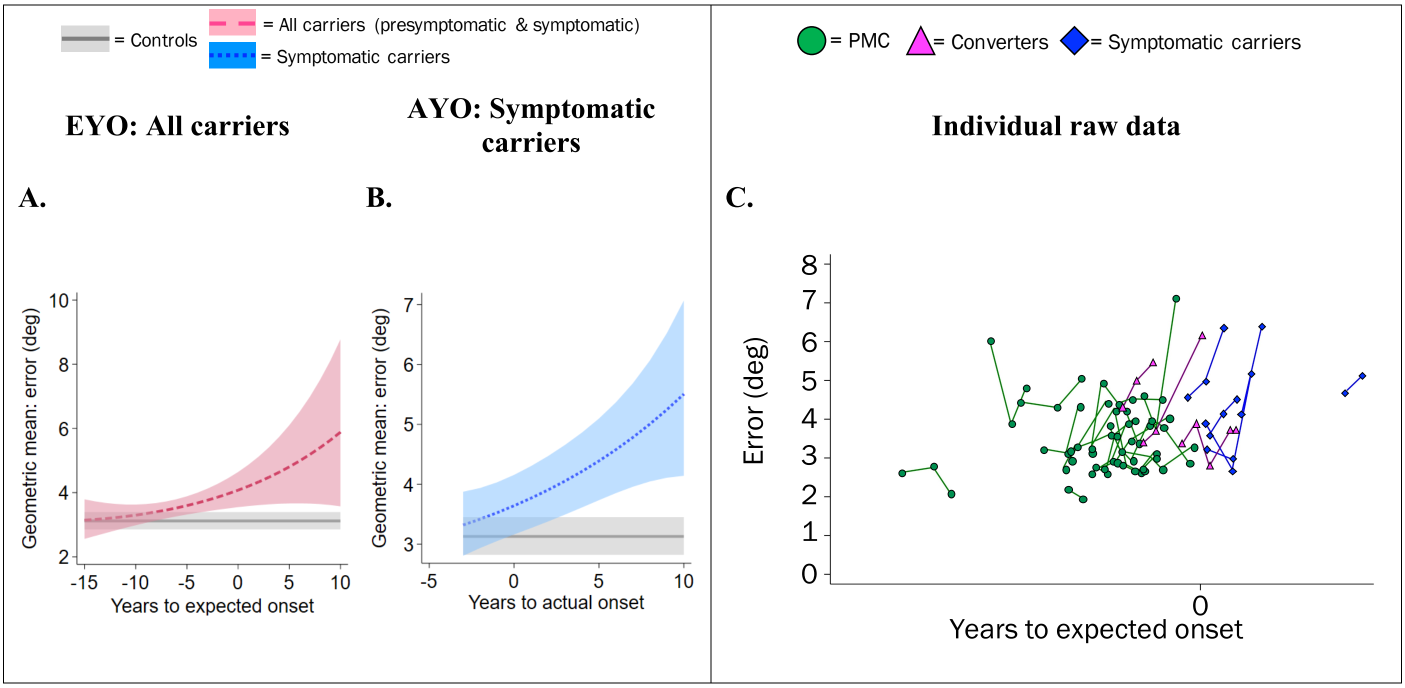


**Figure-e4. Relationship between NIC performance and proximity to symptom onset, 4s delay.** Panels A and B show the predicted NIC mean (from models adjusted for age, sex and NART). **A.** Against EYO. **B.** Against AYO. Shaded area indicates 95% confidence intervals. Panel **C** shows the unadjusted raw NIC data plotted against EYO with visits marked as dots and connected for each participant; note there is no scale on the x-axes to preserve participant anonymity. Converters are PMCs who transitioned into a symptomatic stage at their last visit. NIC= nearest item control; PMC=presymptomatic mutation carrier; EYO=estimated years to/from symptom onset; AYO=actual years to/from symptom onset.

### *2.3. More details on the longitudinal rates of change (N=48)*

#### *2.3.1. Effect of delay, memory load and block on VSTM performance in the longitudinal analysis*

*Delay*: Across all participants, performance was significantly worse for long than short delay for localisation error (24 [95% CI 20, 28] % greater with 4s *vs* 1s, $p<0.001$); NIC (14 [11, 17] % greater with 4s *vs* 1s, $p<0.001$); identification (OR=0.75 [0.67, 0.84] for 4s *vs* 1s, $p<0.001$) and swap error rate (coefficient=0.024 [0.000, 0.047] for 4s *vs* 1s, $p=0.048$).

*Memory load (number of items):* Across all participants, performance significantly worse for high memory-load than low memory load for localisation error (2.3 [1.9, 2.7] times greater with 3 vs 1 item, $p<0.001$) and identification (OR=0.24 [0.19, 0.30] for 3 items *vs* 1 item, $p<0.001$). Swap and localisation error with NIC are only assessed in the 3-items condition.

*Block:* Across all participants, performance was significantly better in block 2 than block 1 for localisation error (12 [10, 15] % smaller for block 2 *vs* block 1, $p<0.001$); NIC (6 [4, 9] % smaller for block 2 *vs* block 1, $p<0.001$) and swap error (coefficient=-0.068 [-0.091, -0.044] for block 2 *vs* block 1, $p<0.001$) and identification also showed a trend for better performance in block 2 (OR=1.12 [1.00, 1.26] for block 2 *vs* block 1, $p=0.055$).

### *2.3.2. Effect of demographic variables on VSTM performance in the longitudinal analysis*

*Age*: Across all participants, there was weak evidence that older age at baseline tended to be associated with greater localisation error (-0.8 [-0.2, 1.9] % change in error per 1 year increase in age, $p$=0.110); greater NIC error (0.7 [0.0, 1.5] % greater error per 1 year increase in age, $p$=0.055); and lower identification performance (OR=0.98 [0.96, 1.00] per 1 year increase in age, $p$=0.070). There was no significant effect of age on swap error rate (regression coefficient=0.001 [-0.001, 0.004], $p$=0.341).

*Sex*: Across all participants, did not show significant associations with localisation error (3.9 [-18.1, 12.8] % for males *vs* females, $p$=0.629); NIC error (-4.9 [-15.2, 6.8] % for males *vs* females, $p$=0.398); identification performance (OR=0.81 [0.58, 1.12] for males *vs* females, $p$=0.200); or swap error rate (regression coefficient=0.026 [-0.021, 0.073] for males *vs* females, $p$=0.279).

*NART:* Across all participants, there was little evidence of an effect of NART on localisation error (-0.4 [-1.3, 0.4] % per point increase, $p$=0.345); NIC (-0.2 [-0.8, 0.4] % per point increase, $p$=0.439); identification performance (OR=1.01 [1.00, 1.03] per point increase, $p$=0.120); or swap error rate (regression coefficient=-0.002 [-0.004, 0.001] per point increase, $p$=0.127).

### *2.3.3. Motor function in the longitudinal analysis*

Following the greater localisation error in late PMCs, we considered whether these differences might be due to motor function difficulties rather than deficits in recall (i.e. participants were less precise at selecting the fractal in the before dragging it to its remembered location). In order to evaluate this, we calculated the distance from the participant selected location within the stimulus to its centre every time a correct fractal was selected in a post-hoc analysis. This was quantified as the deviation from the centre (see Fig.e-3). For early PMCs, there was no evidence of a difference in rate of change of mean deviation from the centre compared to controls (difference in change per year = -2.6 [-8.8, 4.0] %, $p$=0.429). Late PMCs showed a significantly *slower* increase in the deviation from the centre compared to controls (difference in change per year = -7.7 [-13.9, 1.0] %, $p$=0.024). Symptomatic carriers also showed a trend towards a slower increase compared to controls (difference in change per year = -9.1 [-17.5, 0.2] %, $p$=0.054). Delay (regression coefficient = -0.06 [-0.09, -0.03]) and load (regression coefficient= -0.07 [-0.11, 0.03]) also had significant effects on the deviation whereby longer delay and higher load were associated with *smaller* deviation from the centre (both $p$<0.001). While participants were not explicitly asked to select the centre of the stimuli when making a choice, the significant effect of delay and load as well as the smaller deviation for late PMCs compared to controls, suggests that the faster decline in VSTM observed in the localisation metric for late PMCs cannot be entirely explained by a motor impairment, especially as the deviation from the centre was smaller for late PMCs than controls.

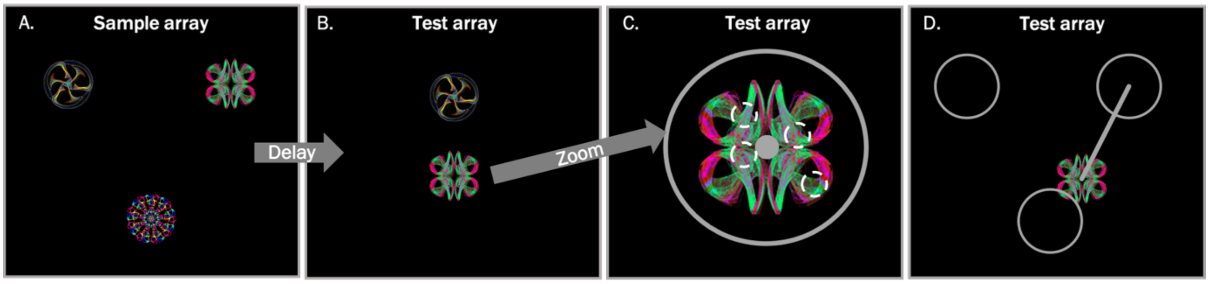


**Figure e3. Illustration of the motor function estimation. A.** Sample array where either 1- or 3- items are presented (3-items in this case). **B.** The participant is asked to make a choice between two fractals where one is the target and the other a distractor or foil. **C.** The deviation from the centre is calculated by measuring the distance within the fractal between the centre of the fractal (grey circle) and the position of the participant's finger once they select the correct fractal (different positions within the fractal are illustrated as white dotted circles). **D.** The localisation error measure where the distance from the centre of the fractal to the participants' chosen location within the array is measured.

### *2.4. Comparison between new participants and those included in Liang and colleagues report (Liang et al. 2016)*

A direct comparison between the new participants and the cohort previously published by Liang and colleagues (Liang et al., 2016) is presented next (Fig-e.5). Notably, this comparison focuses on the two metrics in which presymptomatic participants exhibited deficits in Liang and colleagues report: localisation of the target (specifically 3-items, across delays, block 1) and swap error proportion (specifically 4s delay, block 1).

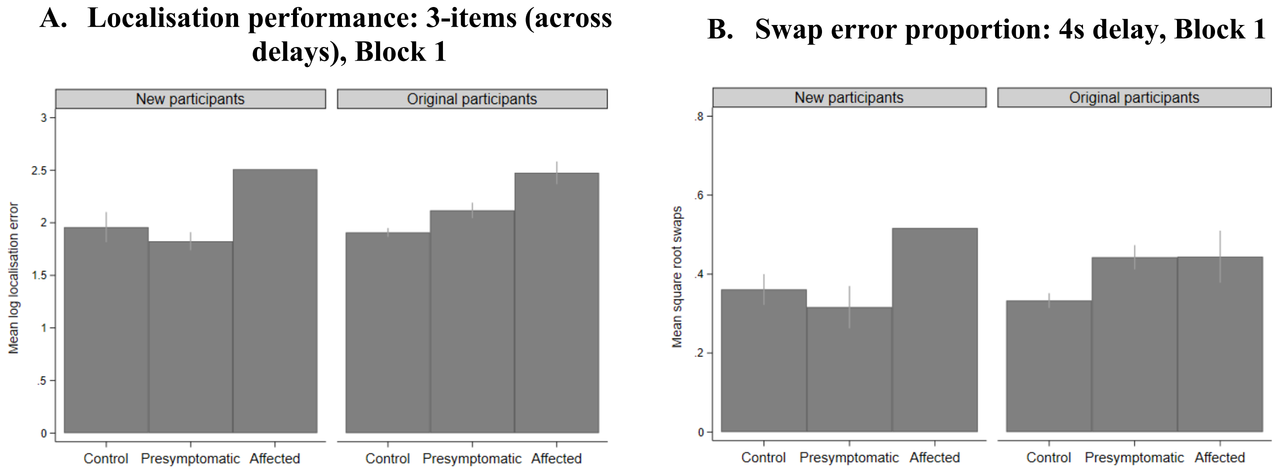


**Figure-e5**. **Mean performance (from model adjusted for age, sex and NART) by group for the 'new participants' added since Liang and colleague's publication compared to the 'original participants' included in that study**. **A.** Localisation performance: 3-items, across delays, block 1. **B.** Swap error proportion: 4s delay, block 1. Group is categorised into: control, presymptomatic and symptomatic or affected to avoid unblinding. Reasons for missing error bar are: 1) the score was for one person; 2) there was no variability in score (e.g. 100% correct).
